# UNC93B1 Mediates Host Resistance to Infection with *Toxoplasma gondii*

**DOI:** 10.1371/journal.ppat.1001071

**Published:** 2010-08-26

**Authors:** Mariane B. Melo, Pia Kasperkovitz, Anna Cerny, Stephanie Könen-Waisman, Evelyn A. Kurt-Jones, Egil Lien, Bruce Beutler, Jonathan C. Howard, Douglas T. Golenbock, Ricardo T. Gazzinelli

**Affiliations:** 1 University of Massachusetts Medical School, Worcester, Massachusetts, United States of America; 2 Institute for Genetics, University of Cologne, Cologne, Germany; 3 The Scripps Research Institute, La Jolla, California, United States of America; 4 Centro de Pesquisas Réne Rachou, Fundação Oswaldo Cruz, Belo Horizonte, Minas Gerais, Brazil; 5 Universidade Federal de Minas Gerais, Belo Horizonte, Minas Gerais, Brazil; Washington University School of Medicine, United States of America

## Abstract

UNC93B1 associates with Toll-Like Receptor (TLR) 3, TLR7 and TLR9, mediating their translocation from the endoplasmic reticulum to the endolysosome, hence allowing proper activation by nucleic acid ligands. We found that the triple deficient ‘3d’ mice, which lack functional UNC93B1, are hyper-susceptible to infection with *Toxoplasma gondii*. We established that while mounting a normal systemic pro-inflammatory response, *i.e.* producing abundant MCP-1, IL-6, TNFα and IFNγ, the 3d mice were unable to control parasite replication. Nevertheless, infection of reciprocal bone marrow chimeras between wild-type and 3d mice with *T. gondii* demonstrated a primary role of hemopoietic cell lineages in the enhanced susceptibility of *UNC93B1* mutant mice. The protective role mediated by UNC93B1 to *T. gondii* infection was associated with impaired IL-12 responses and delayed IFNγ by spleen cells. Notably, in macrophages infected with *T. gondii*, UNC93B1 accumulates on the parasitophorous vacuole. Furthermore, upon *in vitro* infection the rate of tachyzoite replication was enhanced in non-activated macrophages carrying mutant *UNC93B1* as compared to wild type gene. Strikingly, the role of UNC93B1 on intracellular parasite growth appears to be independent of TLR function. Altogether, our results reveal a critical role for UNC93B1 on induction of IL-12/IFNγ production as well as autonomous control of *Toxoplasma* replication by macrophages.

## Author Summary

One third of the human population in the world is chronically infected with *Toxoplasma gondii.* While the majority of infected individuals are asymptomatic, toxoplasmosis is a major cause of congenital disease, abortion, and a life-threatening opportunistic disease in immunocompromised individuals. Early activation of the innate immune system and cytokine production by myeloid cells is required for establishment of protective immunity to *T. gondii* infection. In mice, a mutation in the *UNC93B1* gene abolishes signaling via the intracellular innate immune receptors, namely Toll-like receptors (TLR) 3, 7 and 9, thus, named triple-deficiency (3d) mice. Our results demonstrate that the hyper-susceptibility of 3d mice to *T. gondii* infection is associated with impaired IL-12 production, delayed IFNγ production, and uncontrolled parasite replication in macrophages. Overall, our study reveals a critical role for UNC93B1 in the immunological control of *T. gondii* infection.

## Introduction

*Toxoplasma gondii* is a widespread obligate intracellular protozoan parasite, which establishes itself in the brain and muscle tissues, persisting for life in humans and other vertebrate hosts [1]. One of the most distinctive aspects of *T. gondii* life cycle is the establishment of an often benign chronic infection, which is dependent on the parasite's ability to elicit a strong and persistent cell-mediated immunity [1]. Severe forms of toxoplasmosis are often seen in humans with an immature or suppressed immune system. In particular, cytokines that activate macrophage effector functions, such as IFNγ and TNFα, are critical in mediating host resistance to *T. gondii* infection [2].

The mammalian Toll-like receptors (TLRs) sense conserved molecules from all classes of microorganisms [3], including those from protozoan parasites [4]. Studies employing MyD88^−/−^ mice, which are deficient in the function of most TLRs (except for TLR3), suggest that TLRs are critical in many aspects of host:protozoan parasite interaction, including the initiation of the pro-inflammatory cytokine response and the expression of co-stimulatory molecules [5], [6], [7]. The initial activation of the innate immune system leads to the immediate activation of anti-microbial effector mechanisms. In addition, innate immune activation gives way, over time, to the development of Th1 lymphocytes and host resistance to protozoa, including *T. gondii* [4]. TLR2, TLR4, TLR9, and TLR11 have been shown to be important cognate innate immune receptors involved in the recognition of *T. gondii* derived components, such as glycosilphosphatidylinositol (GPI) anchors [8], CpG DNA [9] and profilin [10], [11]. However, the deficiency of each of these TLRs, and even the loss of two TLRs, as is the case with TLR2/TLR4 double knockout mice, leads to a relatively minor phenotype after *T. gondii* infection, as compared to the results obtained with infected MyD88^−/−^ mice [6].

The “endosomal TLRs”, TLR3, TLR7, TLR8 and TLR9, recognize microbial RNA and DNA [12], [13], [14], [15]. In addition to its well-described function in the recognition of CpG motifs, TLR9 has been shown to play an important role in the recognition of parasite DNA and host resistance to infection by several different protozoan parasites [16], [17], [18]. However, the combined role of nucleotide-sensing TLRs in host resistance to *T. gondii* has not been explored. Tabeta and colleagues [19] identified a mutant mouse line by forward genetic screening that they named “3d”, so called because of its deficiency in response to TLR3, TLR7 and TLR9 ligands (mouse TLR8 do not respond to single stranded RNA). The 3d mouse was shown to have altered UNC93B1 function, an endoplasmic reticulum protein with distant homology to an ion transporter in worms. UNC93B1 is now known to be essential for signaling through mouse TLR3, TLR7, and TLR9, and the consequent production of pro-inflammatory cytokines [19], [20]. The combined deficiency of nucleic acid-sensing TLRs results in altered host resistance to microbial infections [3], [19], [20]. Specifically, UNC93B1 associates and mediates the translocation of the nucleotide-sensing TLRs from the endoplasmic reticulum (ER) to the endolysosomal compartment, allowing their proper activation by microbial RNA and DNA [21], [22].

Here, we show that although mounting a normal systemic pro-inflammatory response, the 3d mice are extremely susceptible to infection with *T. gondii*. Nevertheless, we provide evidence of a critical role of UNC93B1 in mediating IL-12 as well as early IFNγ production during acute infection with *T. gondii*. Its well known that active host cell invasion by *T. gondii* leads to formation of a high pH-parasitophorous vacuole [23], parasite replication and parasitism, whereas passive internalization of the parasite by phagocytosis results in parasite elimination in the lysosomes [24], [25]. We also found that in macrophages infected with *T. gondii*, UNC93B1 translocates to the parasitophorous vacuole, rather than to the predicted phagolysosomes. Finally, our results demonstrate that the lack of functional UNC93B1 results in enhanced tachyzoite replication in macrophages. Altogether, our experiments reveal a role for UNC93B1 on IL-12 production induced by *Toxoplasma* infection, as well as an unprecedented TLR-independent role for UNC93B1 on host cell control of *T. gondii* replication, which combined are of central importance for the *in vivo* resistance to infection with this intracellular protozoan parasite.

## Results

### Extreme susceptibility of 3d mice to acute infection with *T. gondii* is associated with enhanced parasite replication and unimpaired systemic production of pro-inflammatory cytokines

**Figure 1:**
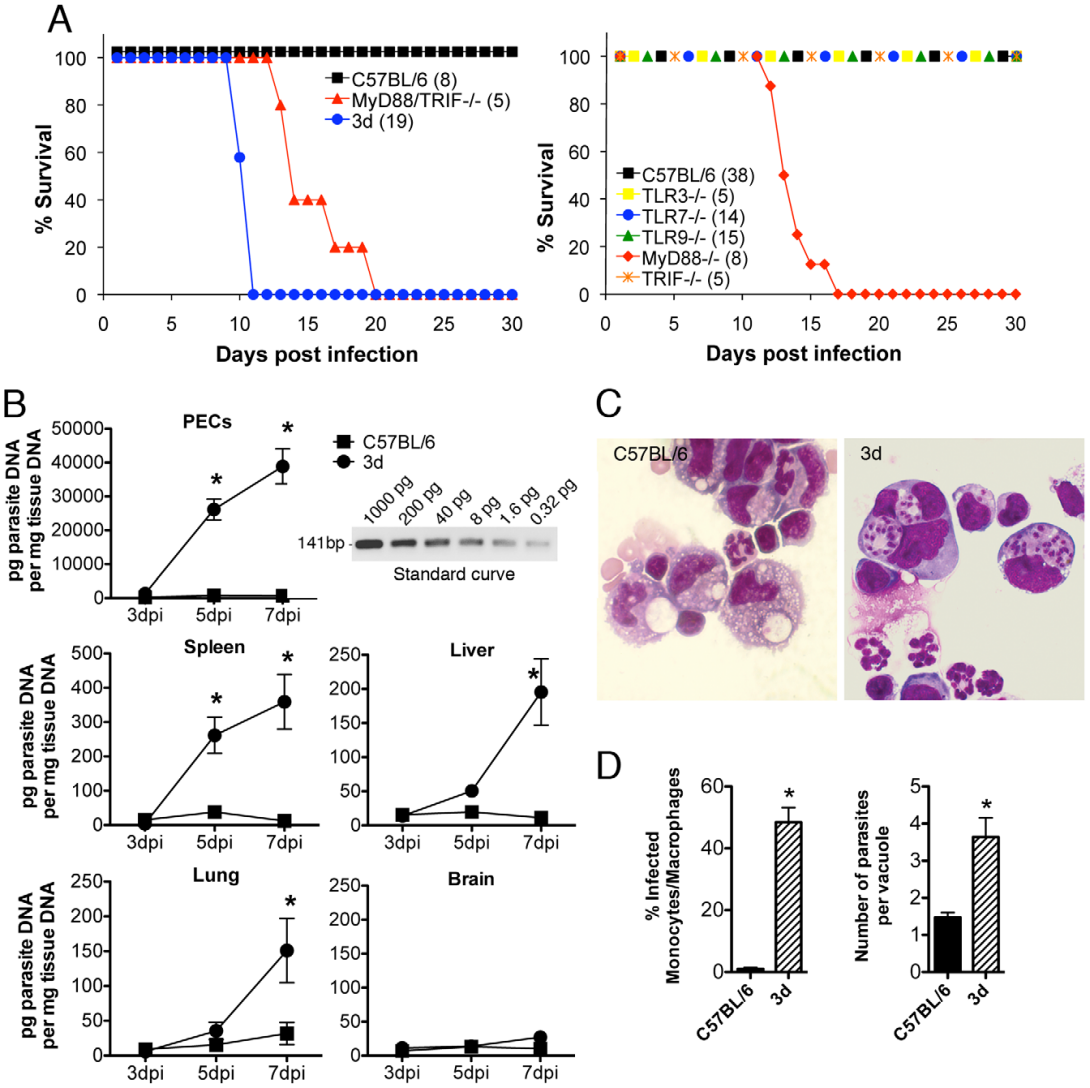
3d mice are highly susceptible to infection with *T. gondii* and succumb due to excessive parasite burden. (A) Mortality after intraperitoneal injection of 25 cysts of the ME-49 strain of *T. gondii*. Data are expressed as percentage of cumulative survival during the experiment. The total numbers of tested animals in each group are indicated within parentheses. *p*<0.0001 when comparing WT versus 3d mice, and *p* = 0.0004 when comparing MyD88KO or MyD88/TRIFdKO versus 3d mice. (B) Quantitative real-time PCR analysis was performed on the indicated tissues collected from animals infected for 3, 5 or 7 days with *T. gondii* cysts. Relative quantification was performed using standard curve analysis of purified parasite DNA. Inset shows an agarose gel electrophoresis of the RT-PCR amplification product obtained using different amounts of purified parasite DNA as template. Results were expressed as pg of parasite DNA per mg of total tissue DNA. Asterisk indicate that *p*<0.05 when comparing WT and 3d mice (error bars, s.e.m.). (C) Photomicrographs of peritoneal exudates stained with Giemsa, collected from C57BL/6 (top panel) or 3d (bottom panel) mice at 8 days after infection with *T. gondii*. (D) Percentage of infected monocyte/macrophage-like cells as well as number of parasites per vacuoles present in the peritoneal exudate cells showed in figure 1C. Asterisk indicate that *p*<0.05 when comparing WT and 3d mice (error bars, s.e.m.).

We found that the 3d mice are highly susceptible to infection with *T. gondii*, suggesting the possibility that the combined action of TLR3, TLR7 and TLR9 is critical for host resistance to infection with *T. gondii*. However, we used as a control the double MyD88/TRIF-null mice, which are deficient in all TLR responses. While very susceptible, we found that upon infection with *T. gondii* the double MyD88/TRIF deficient mice were somewhat more resistant than the 3d mice (Fig. 1A). While this difference was not dramatic, it was consistent, suggesting that UNC93B1 may also mediate host resistance to this parasitic infection by a novel mechanism, independent of TLR function.

**Figure 2:**
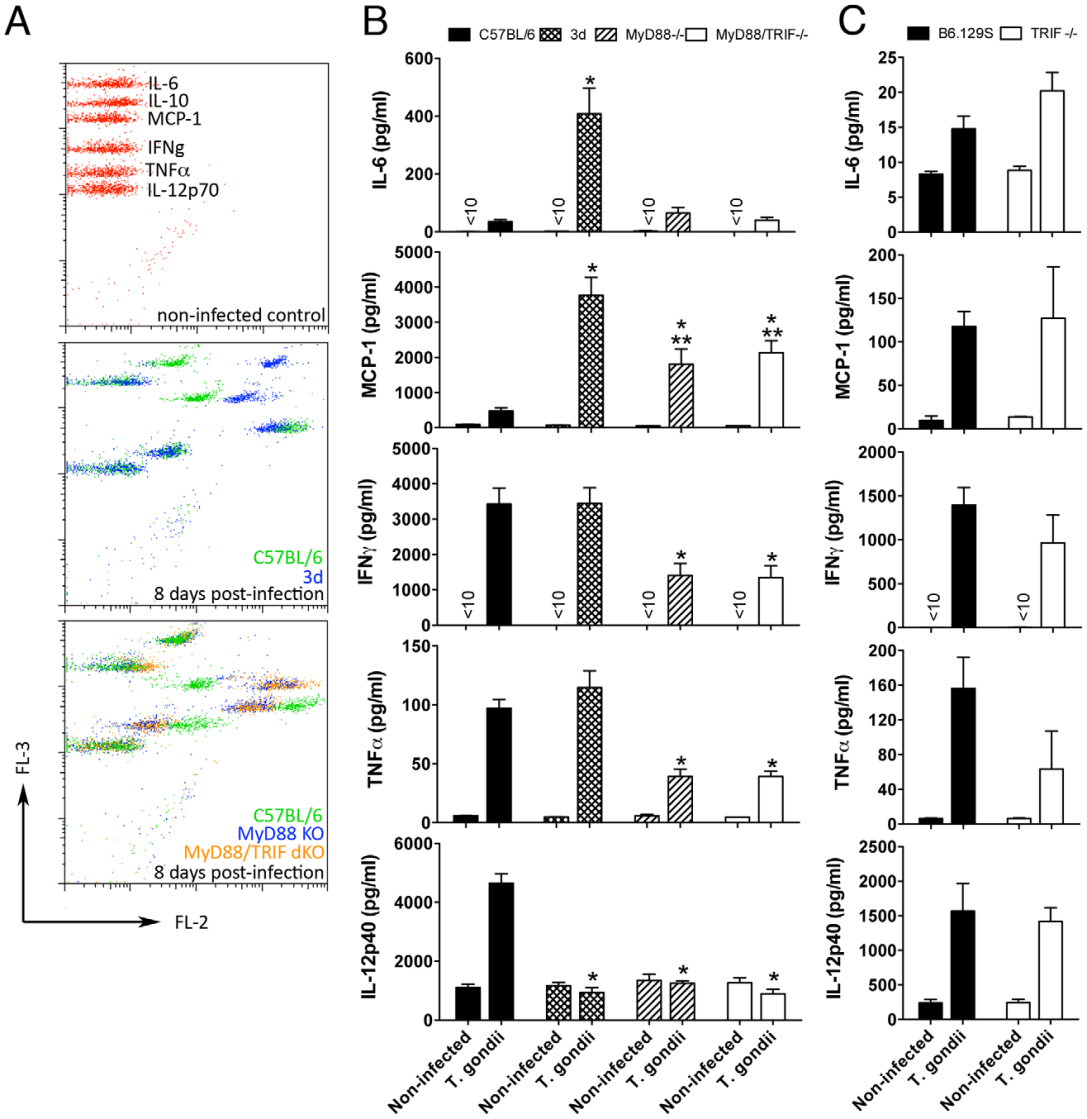
Unimpaired production of pro-inflammatory cytokines in 3d mice infected with *T. gondii*. (A–C) Levels of IL-6, MCP-1, IFNγ, and TNFα measured in sera of mice at 0 and 8 days after infection, employing the BD Cytometric Bead Assay (CBA) Mouse Inflammation Kit. IL-12p40 levels in sera of mice were measured by ELISA. A panel with results from representative individual wild-type, 3d, MyD88-deficient and MyD88/TRIF double deficient animals is shown in A. One asterisk indicates that *p*<0.05 when comparing results from 3d or MyD88-deficient mice to WT animals infected with *T. gondii*. Two asterisks indicate *p*<0.05 when comparing the results from 3d mice with MyD88-deficient animals infected with *T. gondii*. Data are representative of experiments done three (A–B) or two (C) times using at least 4 animals per group (error bars, s.e.m.).

As measured by real-time PCR, enhanced parasite replication was observed in different peripheral organs (*i.e.* spleen, liver, and lungs) (Fig. 1B), despite of unimpaired systemic IFNγ and TNFα production from 3d mice (Figs. 2A and 2B). This is remarkable in view of the long established role for these cytokines in triggering macrophage effector functions and mediating host resistance to *T. gondii*. In agreement with the real-time PCR data, analysis of the cells collected from the peritoneal cavity of infected animals showed significant higher numbers of infected cells in 3d animals (Figs. 1C and 1D). Phenotypic analysis revealed that 55–75% of the cells recruited to the peritoneal cavity of 3d animals expressed the surface marker CD11b. Of these, 75% were neutrophils (CD11b^+^, Ly6G^+^, F4/80^−^) and the remaining 25% were inflammatory monocytes (CD11b^+^, Ly6C^high^, Ly6G^−^, F4/80^low^). Although infected neutrophils were observed, they rarely contained more than two parasites, whereas four or more tachyzoites were typically observed in monocyte/machophage-like cells. Thus, enhanced susceptibility to *T. gondii* infection was associated with uncontrolled parasite replication within monocyte/macrophage-like cells in peritoneal cavity of 3d mice.

In order to further investigate the role of nucleotide-sensing TLRs in resistance to *T. gondii*, we infected the TLR3^−/−^, TLR7^−/−^ and TLR9^−/−^ mice, as well as the single knockout mice deficient in each of the two main adaptors required for TLR function, TRIF and MyD88. Our results showed that except for the MyD88^−/−^ mice, which were very susceptible to infection, all of the other mice lineages had a similar survival curve and cyst numbers, in comparison to wild-type mice (Fig. 1A and data not shown).

We anticipated that the lack of endosomal TLR function would result in impaired cytokine production during infection. However, to our surprise, we found that upon infection with *T. gondii*, except for impaired IL-12p40 production, 3d mice showed high levels of IL-6, MCP-1, IFNγ, and TNFα in their sera (Fig. 2, A–B). Similarly, splenocytes from 3d mice infected with *T. gondii* demonstrated unimpaired *ex-vivo* cytokine production (data not shown). While infected MyD88^−/−^ and MyD88/TRIF double deficient mice had decreased serum levels and *ex-vivo* production of several cytokines (Fig. 2B and data not shown), unimpaired cytokine production of IL-12p40, IFNγ, TNFα, IL-6 and MCP-1 were confirmed in the sera from TRIF^−/−^ (Fig. 2C), TLR3^−/−^, TLR7^−/−^, and TLR9^−/−^ mice (Fig. S1) infected with *T. gondii*. Together, these initial results suggest that the extreme susceptibility of the 3d mouse to *T. gondii* infection is due to an additional function of UNC93B1 that is not related to the regulation of endosomal TLRs.

### Enhanced susceptibility of 3d mice to *T. gondii* infection is mediated by cells from hemopoietic lineage

**Figure 3:**
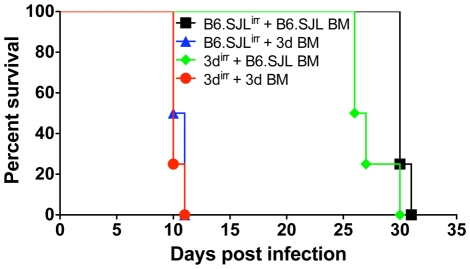
Cells from hemopoietic compartment bearing the *UNC93B1* mutation are responsible for enhanced susceptibility of 3d mice to *T. gondii* infection. B6.SJL-*Ptprc^a^Pepc^b^*/BoyJ (CD45.1^+^) and 3d (CD45.2^+^) mice were used as partners for chimera construction. 6–9 weeks after reconstitution, chimeric mice were infected intraperitoneally with 25 cysts of *T. gondii*, and their survival monitored daily. Data presented are representative of experiments done twice with at least 4 mice per group.

*T. gondii* is a promiscuous parasite that infect any nucleated host cells of both hemopoietic and non-hemopoietic origin. Thus, its replication could be controlled by metabolites secreted by activated macrophages or, alternatively, directly by cytokine-induced microbicidal mechanisms triggered within infected non-phagocytic cells. To distinguish between these two basic mechanisms of cell-mediated immunity, reciprocal bone marrow chimeras were constructed between wild-type and 3d mice and their survival assessed following challenge with *T. gondii*. The reverse chimeras were generated employing wild type (B6.SJL) and 3d mice, which hemopoietic cells express CD45.1 and CD45.2 respectively (Fig. S2A). Notably, transplanted mice, which possess hemopoietic cells from 3d mice, became non-responsive to any of the TLR7 and TLR9 agonists, but sustained cytokine response (TNFα and RANTES) to LPS and Concanavalin A (Fig. S2B). Finally, infectious challenge of reciprocal chimeras demonstrated that expression of wild type, functional UNC93B1, in the hemopoietic, but not in the non-hemopoietic compartment was necessary for host resistance to infection with *T. gondii* (Fig. 3). These findings are consistent with data indicating the primary expression of UNC93B1 in murine cells from myeloid origin (http://biogps.gnf.org/#goto=genereport&id=81622).

### Partial impairment on activation of antigen presenting cells and T lymphocytes from 3d mice

**Figure 4:**
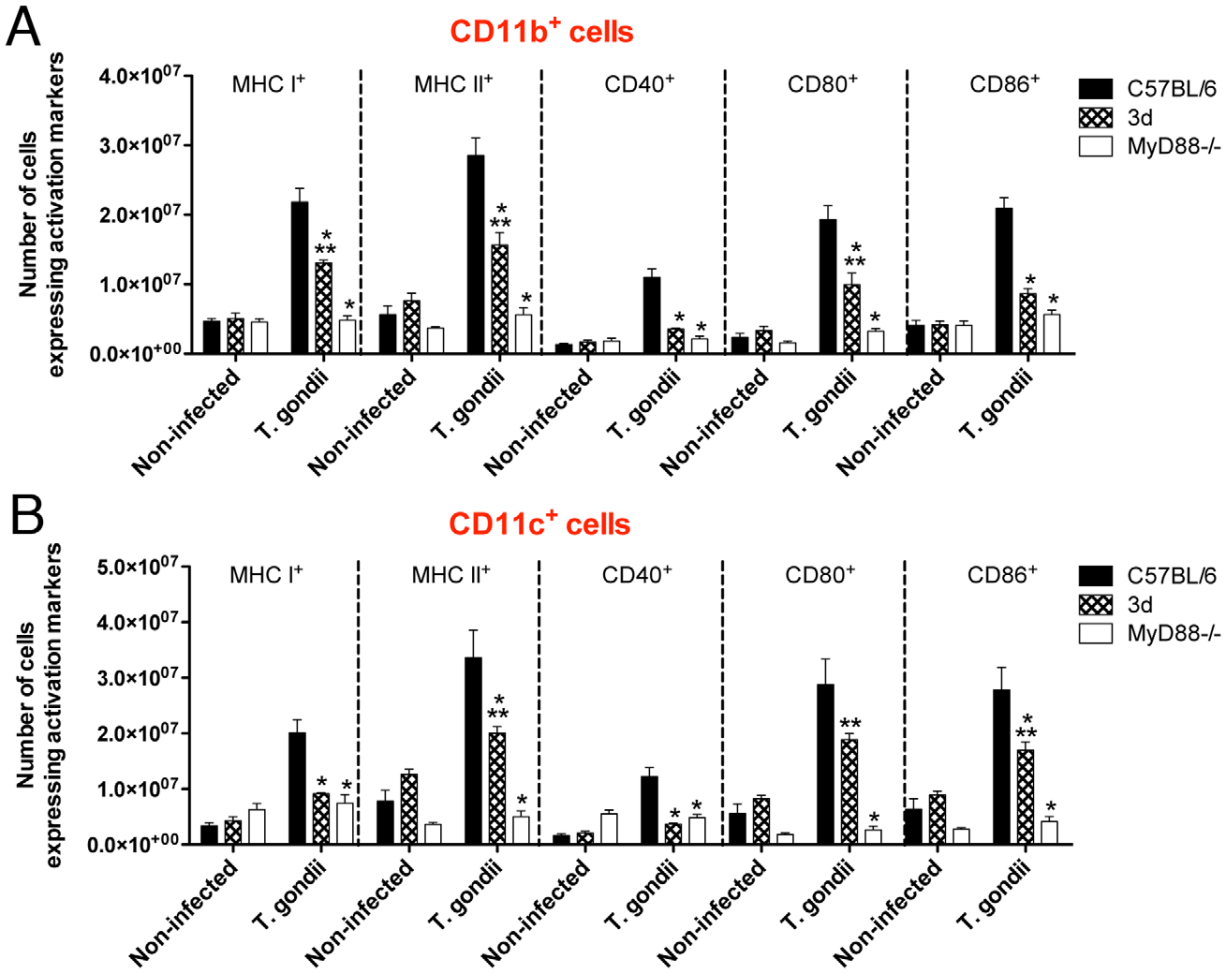
Antigen presenting cells from infected 3d mice express significant amounts of activation markers. (A–B) Flow cytometry analysis of splenocytes isolated from non-infected animals or 8 days after infection with *T. gondii*. Cells were stained with fluorescent antibodies anti-CD11b, anti-CD11c, anti-MHC I, anti-MHC II, anti-CD40, anti-CD80 and anti-CD86. One asterisk indicate that *p*<0.05 when comparing results from 3d or MyD88-deficient mice to WT animals infected with *T. gondii*. Two asterisks indicate *p*<0.05 when comparing the results from 3d mice with MyD88-deficient animals infected with *T. gondii*. Data are representative of experiments done two times, using at least four mice per group (error bars, s.e.m.).

**Figure 5:**
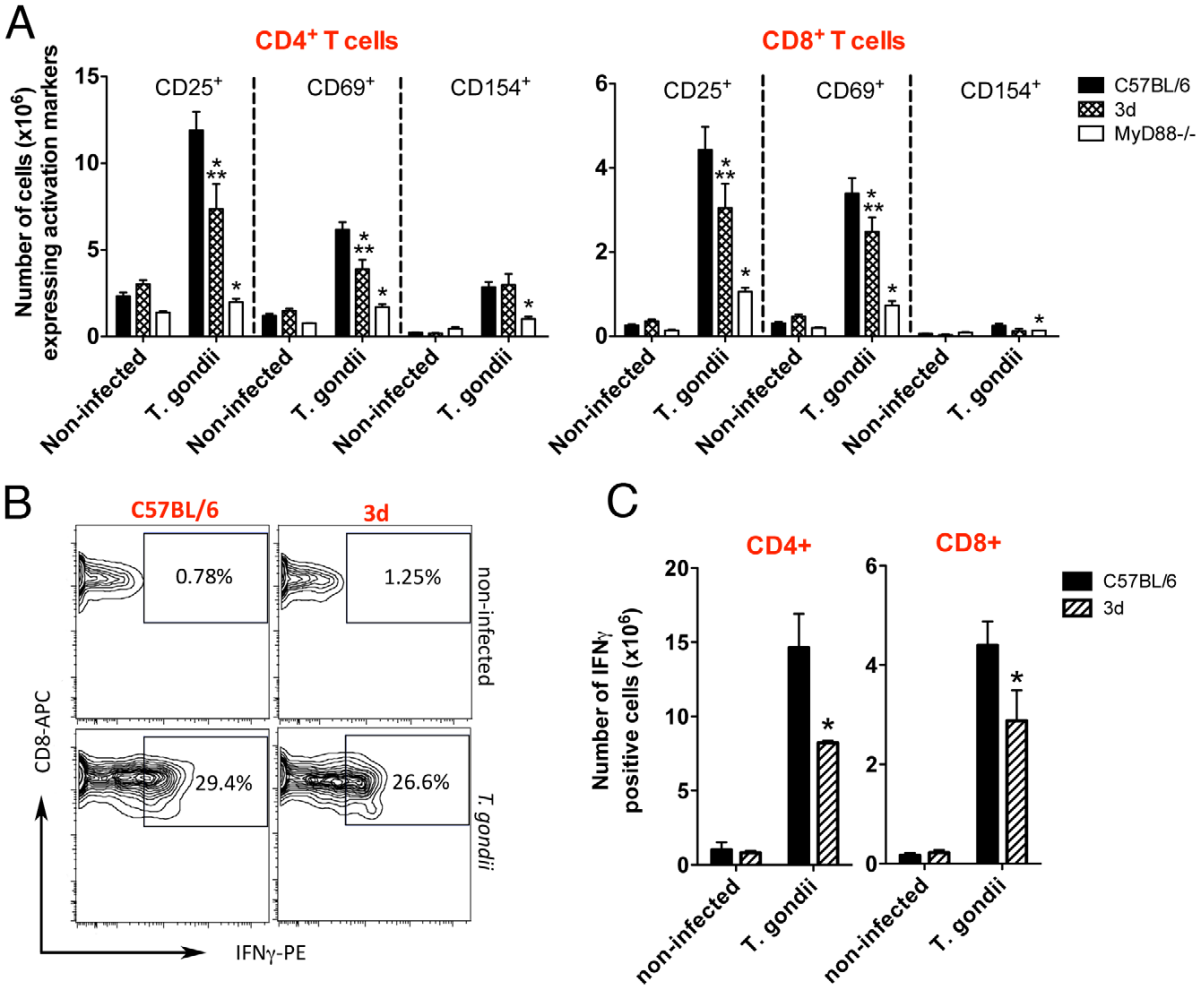
Upon infection with *T. gondii*, 3d mice display a significant number of T cells expressing activation markers in the surface. (A) Flow cytometry analysis of splenocytes isolated from non-infected animals or at 8 days after infection with *T. gondii*. Cells were stained with fluorescent antibodies anti-CD3, anti-CD4, anti-CD8, anti-CD25, anti-CD69 and anti-CD154. (B–C) IFNγ-producing CD4^+^ and CD8^+^-T cells present in splenocytes of C57BL/6 and 3d mice non-infected or at 8 days post-infection, as measured by flow cytometry. A panel with results for IFNγ -positive CD8^+^-T cells from representative individual wild-type and 3d mice is shown in B. One asterisk indicates that *p*<0.05 when comparing results from 3d or MyD88-deficient mice to WT animals infected with *T. gondii*. Two asterisks indicate that *p*<0.05 when comparing the results from 3d mice with MyD88-deficient animals infected with *T. gondii*. Data are representative of experiments done two times, using four mice per group (error bars, s.e.m.).

Based on the results obtained with the reverse chimeras, we decided to focus our attention to evaluate the function of lymphoid/myeloid cells from UN93B1 mutant mice. Because UNC93B1 has also been suggested to be involved on antigen presentation and T cell responses [19], we evaluated the expansion and expression of activation markers on antigen presenting cells (*i.e*., CD11b^+^ and CD11c^+^), as well as CD4^+^ T and CD8^+^ T cells from mice infected with *T. gondii*. CD11b^+^ as well as CD11c^+^ cells from 3d mice expressed significant amounts of activation markers, *e.g.* MHC class I and II, CD40, CD80 and CD86, which were intermediary between cells from wild type and MyD88^−/−^ mice infected with *T. gondii* (Fig. 4). In addition, spleens of 3d mice infected with *T. gondii* contained 20–40% less CD4^+^ and CD8^+^ T cells expressing CD25, CD69 and CD154 (Fig. 5A). While these differences in wild-type vs. infected 3d mice were statistically significant, the impairment in activation of cells was far more pronounced in MyD88^−/−^ mice (Fig. 4 and Fig. 5A), whose splenocytes showed no signs of expansion after *T. gondii* infection (data not shown). The percentage of CD4^+^T as well as CD8^+^T lymphocytes producing IFNγ was similar when comparing wild type and 3d mice (Fig. 5B), whereas the total number of IFNγ -producing T cells was significantly smaller in infected 3d as compared to wild type mice (Fig. 5C).

### Normal IFNγ responsiveness in 3d mice infected with *T. gondii*

**Figure 6:**
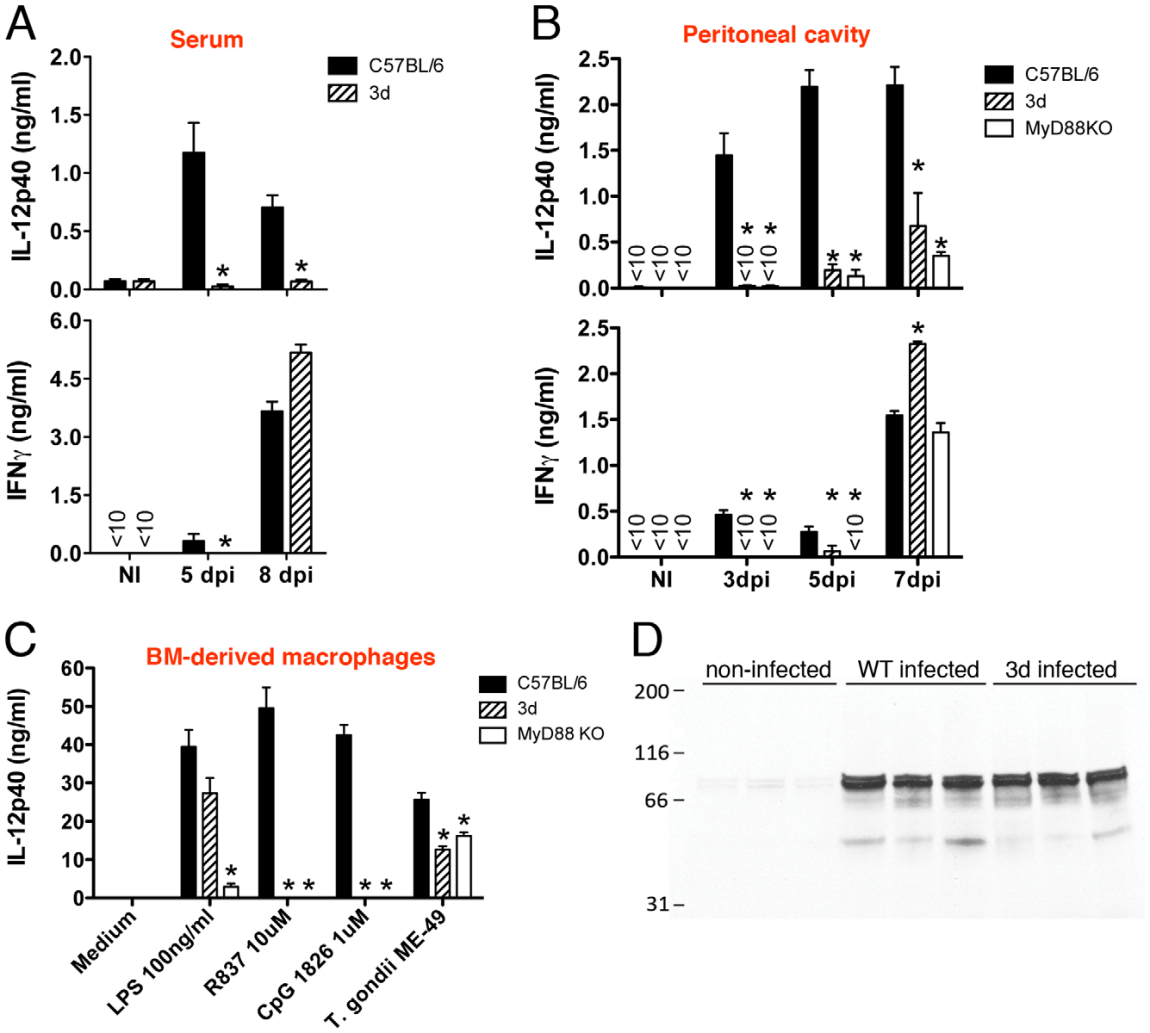
UNC93B1 mutation affects IL-12 and early IFNγ production. (A) Levels of IL-12p40 and IFNγ produced by splenocytes collected from non-infected controls, as well as mice at 5 and 8 days after infection with *T. gondii*. Spleen cells were cultured for 24 h in absence of exogenous stimuli. (B) Levels of cytokines present in the peritoneal cavity exudate of non-infected controls, as well as mice at 3, 5 or 7 days after intraperitoneal infection with *T. gondii* cysts. (C) Wild-type, 3d and MyD88 knockout bone marrow-derived macrophages were stimulated overnight with various TLR agonists or infected with *T. gondii* (MOI 5∶1), and cytokine levels in supernatants quantified by ELISA. (D) Immunoblot analysis of STAT-1 phosphorylation in splenocytes collected from animals either non-infected or at 8 days after infection. Asterisk indicates that *p*<0.05 when comparing results from 3d or MyD88^−/−^ to WT mice/cells. Data are representative of three (A–C) or two (D) experiments yielding similar results (error bars, s.e.m.).

Consistent with the low serum levels of IL-12 (Fig. 2) and partial impairment on activation of antigen presenting cells (CD11b^+^ or CD11c^+^ cells) (Fig. 4), we observed a largely impaired IL-12 production by spleen cells from infected 3d mice (Fig. 6A, top panel). Production of IL-12 was also severely impaired at the infection site (Fig. 6B, top panel). Importantly, bone-marrow derived macrophages from 3d animals exposed to *T. gondii* tachyzoites *in vitro* also produce 50% less IL-12 than WT cells (Fig. 6C). Consistent with the delayed production of IL-12, we also observed a late IFNγ response by spleen or peritoneal exudate cells from 3d mice, which was lower on days three and five, but not on days seven or eight post-infection (Figs. 6A and 6B, bottom panels).

IFNγ is thought to be the most critical cytokine in controlling *T. gondii* replication [2], and mediates the IL-12 role on host resistance to *T. gondii* [26], [27]. Despite the fact that MyD88 knockout and MyD88/TRIF double deficient animals displayed a similar impairment on IL-12 production as the 3d mice (Figs. 2B and 6B–C), and an even more dramatic defect on IFNγ production (Fig. 2B, and 6B–C), the 3d mice consistently showed a more pronounced susceptibility to *T. gondii* infection (Fig. 1A). Therefore, we believe that the delay of IFNγ production could not be solely responsible for the profound susceptibility phenotype observed on 3d mice. Thus, we sought to determine if cells from 3d mice were properly responding to IFNγ. We observed normal STAT1 phosphorylation, an essential component for IFNγ signaling, in 3d mice infected with *T. gondii* (Fig. 6D). Furthermore, 3d macrophages responded normally to IFNγ by producing high levels of TNFα, IL-12, and IL-6 when stimulated *in vitro* in combination with LPS (Fig. S3). Similarly, activation with IFNγ resulted in the enhanced expression of MHC I, MHC II and CD40 in macrophages from 3d mice (Fig. S3). Thus, we found no evidence that the response to IFNγ is affected in mice lacking functional UNC93B1.

### UNC93B1 accumulates around the parasitophorous vacuole in host cells infected with *T. gondii*

Since we found large amounts of parasite within the peritoneal cavity of 3d mice, despite of the high levels of IFNγ, we decided to investigate the ability of macrophages expressing UNC93B1 to control tachyzoite growth *in vitro. T. gondii* can actively invade host cells or it can be actively internalized by professional phagocytic cells. The fate of the parasite inside the host cell depends on the way the parasite is internalized [24], [28]. Active invasion normally results in generation of a unique organelle known as the parasitophorous vacuole, which is incompetent to fuse with lysosomes. The parasitophorous vacuole consequently has a high pH and is not stained by LysoTracker [23]. In contrast to active invasion by the parasite, phagocytosis directs the parasite to the phagolysosome, which has a low pH, and leads to elimination of the parasite.

**Figure 7:**
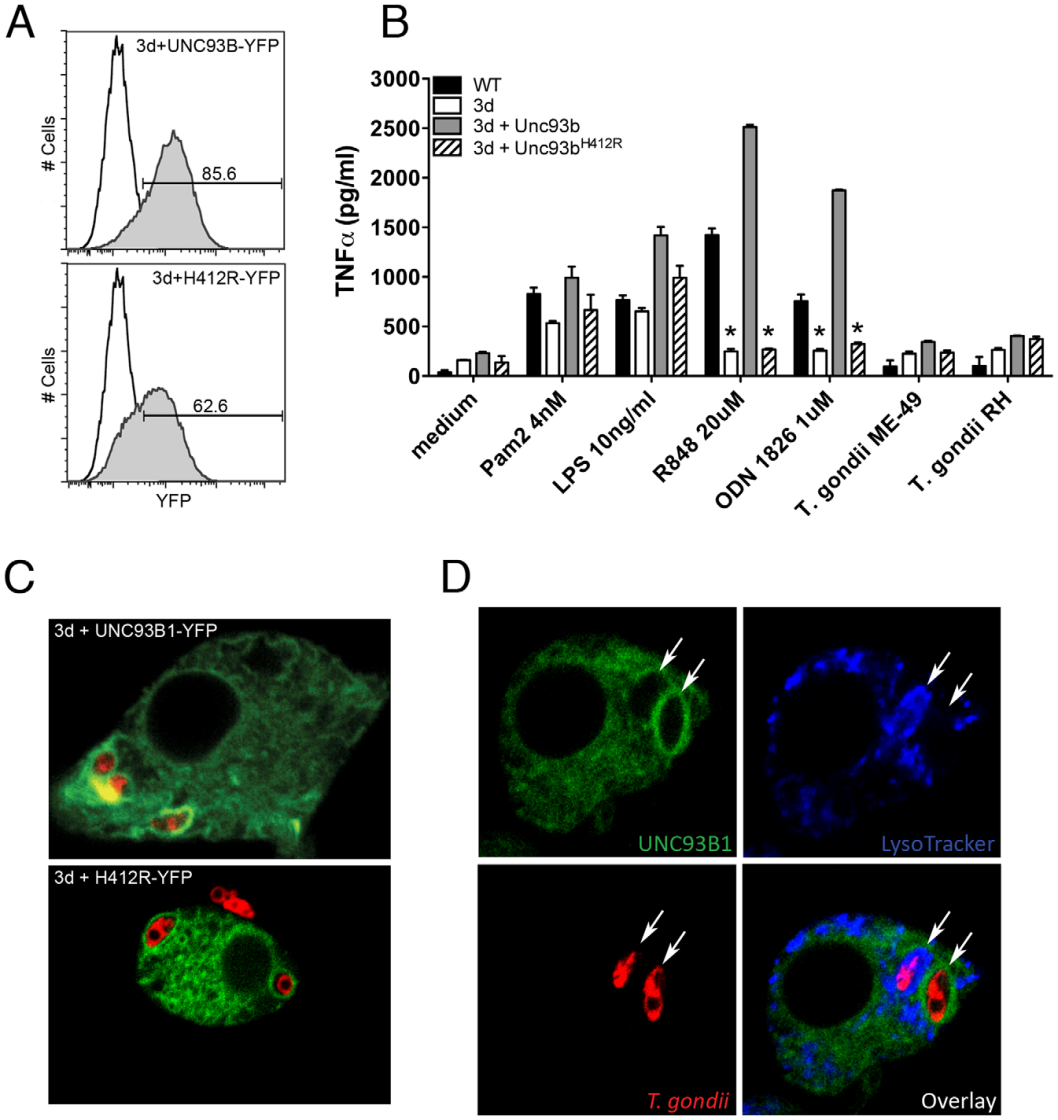
UNC93B1 accumulates around *T. gondii* parasitophorous vacuole. (A) Flow cytometry analysis of immortalized macrophages from 3d mice transduced with YFP-tagged wilde-type or mutant UNC93B1. Black line and shaded histograms show the results from non-transduced controls and cells transduced with UNC93B1-YFP, respectively. (B) Wild-type, 3d immortalized macrophages as well as 3d macrophages stably expressing wild-type or mutated (H421R) form of UNC93B1 were stimulated overnight with various TLR agonists or infected with *T. gondii* (MOI 5∶1). TNFα production was assessed by ELISA in cell culture supernatants. Asterisk indicates that *p*<0.05 when comparing results to WT cells. (C–D) Confocal microscopy of immortalized macrophages from 3d mice stably expressing wild type or mutant UNC93B1-YFP and infected with CMTPX-stained *T. gondii*. Live cells were imaged 2 h after infection. Acidic compartments were stained with LysoTracker Blue. 92% of tachyzoites present in LysoTracker negative compartments were surrounded by a membrane enriched with YFP-tagged UNC93B1. Arrows indicate internalized parasites. Data are from one representative experiment of two (A–B) or four (C–D).

We next evaluated the association of UNC93B1 with *T. gondii* parasites in infected host cells. We began by generating immortalized macrophage cell lines from the UNC93B1 mouse. These lines were then genetically engineered to express either YFP-tagged wild-type UNC93B1 or YFP-tagged UNC93B1^H412R^ (the non-functional mutant expressed by the 3d mouse). Both cell lines expressed high levels of UNC93B1 (Fig. 7A). Macrophage cell lines expressing the wild-type form, but not the mutated UNC93B1^H412R^, recovered cytokine responses to agonists for TLR9 and TLR7, respectively (Fig. 7B). In non-activated cells, UNC93B1 was observed as a resident ER protein, and did not co-localize to LysoTracker positive acidic compartments (Fig. S4). Confocal microscopy revealed that after infection, there was an enrichment of UNC93B1 around the internalized parasites in cell lines that expressed the wild-type, but not the mutated/non-functional protein (Fig. 7C).

The results presented in Fig. 7D show a macrophage cell containing two intracellular parasites. One of these parasites was seen in the acidic LysoTracker positive phagolysosome, and was not surrounded by UNC93B1. Conversely, the parasite found in the parasitophorous vacuole was LysoTracker negative and was surrounded by an intense green ring, indicating a high concentration of UNC93B1-YFP around the parasitophorous vacuole, but not in the predicted phagolysosomes [22]. Immunofluorescence analysis of the parasitophorous vacuoles showed that 92% of internalized tachyzoites present in LysoTracker negative compartments, also positive for the parasite-specific protein GRA7 [29], were surrounded by a membrane enriched with UNC93B1. Such enrichment was never observed in acidic organelles containing phagocytosed parasites.

### UNC93B1 promotes host cell resistance to infection with *T. gondii* parasites

**Figure 8:**
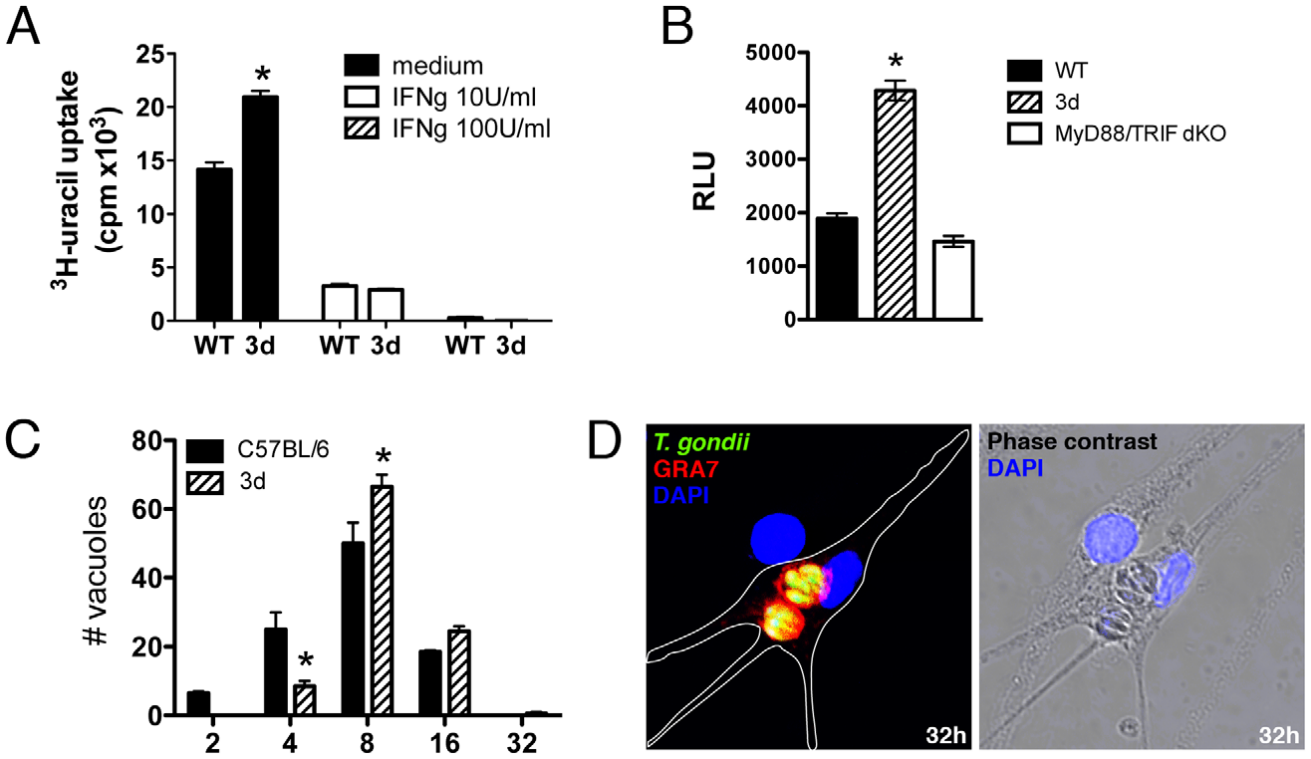
UNC93B1 mediates host cell resistance to infection with *T. gondii* through an IFNγ–independent mechanism. (A) Immortalized macrophages were treated overnight with indicated concentrations of IFNγ, and subsequently infected with ME-49 tachyzoites in a multiplicity of infection of 1. The growth of intracellular parasites was monitored by uracil incorporation assay. (B) Immortalized macrophages from WT, 3d and MyD88/TRIF double deficient mice were infected for 48 h with a 1∶1 ratio of luciferase expressing tachyzoites per cell and the relative luciferase units (RLU) were calculated by normalizing the raw luminescence values to the background (error bars, s.e.m.). Asterisk indicates that *p*<0.05 when comparing results from 3d to WT or MyD88/TRIF^−/−^ cells. (C–D) BMDMs isolated from WT or 3d mice were infected with GFP-expressing parasites at a multiplicity of infection of 1, and the number of parasites per vacuole was quantified 32 hours post infection. To differentiate parasites in phagosomes from parasites in the PV only vacuoles positive for GRA7 were counted. Absolute numbers are shown. Representative images of infected 3d BMDMs is shown in D. Asterisk indicates that *p*<0.05 when comparing results to WT cells. Data are representative experiment of three.

We also tested the ability of bone marrow immortalized macrophages derived from wild type and 3d mice to control parasite replication *in vitro*. Our results show that upon activation IFNγ (10 or 100 U/ml) macrophages from 3d mice were as efficient as the ones derived from wild type mice to control tachyzoite replication as detected by ^3^H-uracil uptake (Fig. 8A). Notably, the levels of reactive nitrogen intermediates release as measured by nitrite levels (Fig. S5A), induction of Irga6, Irgm1 and Irgm3 (Fig. S5B), and translocation of Irga6 and Igrb6 to the parasitophorous vacuole (Fig. S5C), all involved on tachyzoite control by IFNγ activated macrophages, were normal in cells from 3d mice. Nevertheless, we consistently observed and enhanced replication of tachyzoites in non-activated macrophages from 3d mice as compared to the same cells derived from wild type mice. Thus, we further explore the possibility that non-activated macrophages from 3d mice are more permissive to tachyzoite growth.

Unprimed immortalized macrophages from wild type, 3d and double MyD88/TRIF knockout mice were infected with *T. gondii* (ME-49 strain) expressing luciferase reporter gene at a ratio of one parasite per cell, and parasite growth evaluated 48 hs later, by measuring luciferase activity (Fig. 8B). Our experiments demonstrated that parasite replication was more pronounced in cells from 3d mice (Fig. 8B). In contrast, when we compared parasite replication in macrophages from wild type versus MyD88/TRIF double knockout mice, no difference was observed in parasite growth (Fig. 8B). Finally, we performed a detailed analysis of parasite growth in bone marrow-derived macrophages from wild type and 3d mice, evaluating the numbers of vacuoles per cells and tachyzoites per parasitophorous vacuole (Figs. 8C and 8D). The results of this experiment demonstrate a lower number of parasitophorous vacuoles containing 2 or 4 tachyzoites (*p*<0.05), and a significantly higher number of vacuoles containing 8 (*p*<0.05) or 16 parasites when comparing macrophages from wild type and 3d mice. The number of parasitophorous vacuoles per infected cell was similar (1.72±0.22) in both cell types. Altogether, these results indicate that UNC93B1 mediates host cell resistance to *T. gondii* through a mechanism that controls parasite replication in the parasitophorous vacuole and is independent from both TLR activation and the anti-parasitic effects of IFNγ.

## Discussion

UNC93B1 is a critical mediator of the translocation of nucleotide-sensing TLR3, TLR7 and TLR9 from the ER to endolysosomes [22]. Here, we tested the 3d mouse, which has a non-functional UNC93B1 [19], to evaluate the combined role of nucleotide sensing TLRs in controlling initial activation of innate immunity and host resistance to infection with *T. gondii*. Despite the fact that none of single TLR3, TLR7 or TLR9 knockout yield an altered phenotype on cytokine response or enhanced susceptibility, we found that 3d mice are extremely susceptible to infection with *T. gondii.* Therefore, our results raise the possibility that combined action of nucleotide sensing TLRs is critical for host resistance to *T. gondii*.

Much to our surprise, the MyD88/TRIF null mice, which are devoid of all TLR functions and show impaired production of pro-inflammatory cytokines when infected with *T. gondii*, were consistently more resistant to infection than the 3d mice. Furthermore, except for IL-12, 3d mice infected with *T. gondii* mounted a normal systemic pro-inflammatory response, while the MyD88/TRIF double knockouts did not, indicating that the immunological response to infection was fundamentally different. Nevertheless, animals bearing UNC93B1 mutation succumbed to infection as a result of unchecked tachyzoite replication, similar to IFNγ^−/−^ mice [30]. Thus, while we cannot exclude that the combined action of intracellular TLR 3, 7 and 9 contributes to host resistance against *T. gondii*, our hypothesis is that UNC93B1 also mediates host resistance against *T. gondii* through an additional mechanism, which is TLR-independent.

Considering the UNC93B1 involvement on antigen presentation [19], we first investigated whether antigen presenting cells (APCs), CD4^+^ T, and CD8^+^ T lymphocytes were properly activated in 3d mice. Our results show that production of IL-12 as well as expression of activation markers by APCs was significantly impaired in 3d mice infected with *T. gondii.* Intriguingly, the IFNγ levels were similar in the sera, splenocyte cultures and peritoneal cavity, when comparing 3d and WT mice at day 8 post-infection with *T. gondii*. While the percentage of T cells producing IFNγ in splenocytes from infected 3d was comparable to infected C57BL/6 mice, the total numbers of IFNγ producing-CD4+ T as well as -CD8+ T cells were lower in de 3d mice. Regardless, mice deficient in CD8^+^ T cells or in the so-called transporter associated with antigen processing (TAP-1) protein, while more susceptible to infection with *T. gondii*, often survive 30–40 days post-infection [31], [32]. Thus, our data suggest that defective antigen cross-presentation and CD8^+^ T cell activation are not the primary events accounting for the extreme susceptibility of 3d mice to *T. gondii* infection.

Importantly, our *in vivo* experiments suggest that UNC93B1 is an important mediator of IL-12 production during *T. gondii* infection. We also addressed this question *in vitro* and observed that exposure of macrophages from 3d or MyD88^−/−^ mice to live ME49 tachyzoites resulted in impaired production of IL-12, as compared to macrophages from WT mice. Since IL-12 is a key mediator of IFNγ production during *T. gondii* infection [26], [27], [33], [34], it is surprising that IFNγ responses, as discussed above, were close to normal in the infected 3d mice. Therefore, we performed experiments at earlier time points and observed that production of IL-12 and IFNγ was significantly impaired in the peritoneal cavity and peritoneal cavity/spleens from 3d mice at 3 and 5 days post-infection, respectively. These findings could be explained as a result of combined deficiency of nucleotide sensing-TLRs, since no phenotype is observed in each of the single TLR3, TLR7 or TLR9 knockout mice.

Consistently, experiments performed in our laboratory and elsewhere [35] demonstrate that despite of severe impairment IL-12 production in MyD88^−/−^ mice, IFNγ is still produced at 8 days post-infection, and yet, mice are highly susceptible to ME-49 infection. Similarly, different studies demonstrate that except for IL-12, infection with the highly virulent RH strain of *T. gondii* elicits elevated levels of pro-inflammatory cytokines, including IFNγ [36], [37]. However due to the inherent ability of RH parasites to rapidly replicate and disseminate, infected animals are still unable to control parasite burden and die during the acute phase of infection [36], [37].

Even though the observed defect on IL-12/IFNγ axis seems to be sufficient to render animals more susceptible to *T. gondii*, our results clearly indicate that an additional TLR independent-function mediated by UNC93B1 contributes for the extreme susceptibility of 3d mice, when compared to MyD88^−/−^ or MyD88/TRIF double knockouts infected with *T. gondii*. Notably, from *in vitro* and *in vivo* experiments we had no evidence that cells from 3d mice have a defect in responding to IFNγ. Thus, we next investigated the ability of host cells from UNC93B1 mutant mice to control parasite replication. The fate of *T. gondii* inside the host cell relies upon the mechanism of entry. Passive internalization by phagocytosis directs parasites to the lysosomal compartment, leading to tachyzoite elimination [24], [25]. Upon active invasion, *T. gondii* establishes itself in the parasitophorous vacuole [38], [39], [40], which avoids fusion with lysosomes, allowing parasite survival. Interestingly, we found that UNC93B1 is recruited from the ER to the parasitophorous vacuole (PV), rather than to the expected endolysosomal compartment [22].

In spite of being considered a non-fusogenic compartment, few host cell proteins are found at the membrane of parasitophorous vacuole containing tachyzoites. Specific proteins appear to be selectively recruited from the host cell plasma membrane [41], or after host cell activation [42]. In addition, the ER is known to be in close contact with the parasitophorous vacuole membrane [43], and fusion between the ER and parasitophorous vacuole containing live parasites has been demonstrated [44]. Notably, UNC93B1 was shown to translocate from ER to the endolysosomal compartment upon cell stimulation with TLR agonists [22]. Notwithstanding, here we show that UNC93B1 is recruited from the ER to the parasitophorous vacuoles. It is likely that the recruitment of UNC93B1 to the parasitophorous vacuole membrane occurs during the process of ER fusion, given that UNC93B1 is an ER resident protein [19], [21]. The transfer of ER proteins to the parasitophorous vacuole membrane seems to be selective, since neither the mutant form of UNC93B1 nor TLR9 (not shown) were found to be enriched around the parasitophorous vacuole. Dissociation of the intracellular traffic of UNC93B1 and TLR9 has also been suggested by Ewald and co-workers [45], as they observed that forced expression of UNC93B1 at the plasma membrane was not accompanied by relocation of TLR9. Certainly, the process that involves selection or exclusion of specific host proteins, including UNC93B1 and nucleotide sensing TLRs, to the parasitophorous vacuole membrane, is likely to be a key event in the successful establishment of parasitism, and remains to be elucidated.

Most of the mechanisms involved in the control of intracellular replication of *T. gondii* have been studied in IFNγ-activated macrophages. For example the downstream effects of GTPases [46], [47], production of reactive nitrogen intermediates [48], tryptophan degradation in human cells [49] or autophagy [50], [51] are all IFNγ-inducible mechanisms involved in controlling and/or killing of *T. gondii* replication. Markedly, we found that *in vitro*, macrophages from 3d mice present a normal response and are perfectly able to control tachyzoite replication when activated with IFNγ. Consistently, the production of reactive nitrogen intermediates as well as and expression and translocation of IFNγ-inducible GTPases (*i.e.* Irga6, Irgb6, Irgm1 and Irgm3) or formation of autophagic vacuoles (data not shown) are not impaired in macrophages from 3d mice activated with IFNγ.

Remarkably, our results demonstrate an uncontrolled parasite replication in macrophages from 3d mice infected *in vivo* with *T. gondii*, despite an unimpaired IFNγ response. Further, we found that *in vitro* the 3d mutation renders non-activated macrophages more susceptible to intracellular tachyzoite replication. Even though the difference in parasite numbers is modest, when comparing macrophages from WT and 3d mice, it may reflect large differences *in vivo*, where multiple rounds of parasite replication during a long period of time will result in exponential parasite growth. To support our interpretation, other studies also show that small but significant differences in parasite replication *in vitro,* reflects dramatic differences in parasite growth and virulence *in vivo* [52], [53]. It is difficult to imagine how this phenomenon could be related to the effects of the UNC93B1 mutation on TLR signaling, since the rates of parasite replication in non-activated macrophages from MyD88/TRIF null mice were similar to that observed in the same cells derived from wild-type mice. Thus, UNC93B1 effects on parasite control appear to be independent of what are thought important immune mediators of host resistance to *T. gondii*, such as TLRs, IFNγ and TNFα.

To establish itself inside a host cell *T. gondii* has to acquire metabolites from intracellular stores. Indeed, UNC93B1 is a distant ortholog to an ion transporter from *Caenorhabditis elegans* [54], [55], [56]. Despite this homology, a similar function has not been described for UNC93B1 in mammals [19]. Alternatively, nutrient can be acquired from channels present at the parasitophorous vacuole membrane, which allow free diffusion of small metabolites up to 1300 Da [57]; lipids may be acquired through the closely apposed mitochondrial and ER membranes [43]; and parasite seems to exploit the host endolysosomal system via sequestration of host organelles into invaginations present at the parasitophorous vacuole membrane [58]. Therefore, it is also possible that UNC93B1 regulates metabolite/nutrient acquisition by *T. gondii* tachyzoites, and hence interferes with parasite replication.

In conclusion, our study reveals a critical anti-parasitic role for UNC93B1. This role appears to involved in at least two steps: (i) control of IL-12 and early IFNγ response, which may be a result of combined TLR3/TLR7/TLR9 deficiency; and (ii) UNC93B1 enrichment in the membranes surrounding the parasitophorous vacuole containing *T. gondii* tachyzoites, which mediates control of parasite growth in a TLR- and IFNγ-independent manner. Altogether our results indicate that UNC93B1 plays a critical role on innate immune response and host resistance to *T. gondii* infection.

## Methods

### Ethics statement

All experiments involving animals were in accordance with guidelines set forth by the American Association for Laboratory Animal Science (AALAS). All protocols developed for this work were approved by the Institutional Animal Care and Use Committee (IACUC) at the University of Massachusetts Medical School.

### Reagents

All cell culture reagents were obtained from Mediatech, unless otherwise indicated. LPS derived from Escherichia coli strain 0111:B4 was purchased from Sigma and re-extracted by phenol chloroform to remove lipopeptides as described [59]. Pam_2_CysSer(Lys)_4_ was obtained from EMC Microcollections. R848, a synthetic small molecule agonist for TLR7 was provided by 3 M Pharmaceuticals. Phosphorothioate-stabilized unmethylated DNA oligonucleotide-bearing CpG (ODN 1826, 5′-TCCATGACGTTCCTGACGTT-3′) and qPCR primers were obtained from Integrated DNA Technologies. Interferon- γ was purchased from R&D Systems.

### Mice

C57BL/6J (CD45.2^+^), B6.129SF2/J (CD45.2^+^) and B6.SJL-*Ptprc^a^Pepc^b^*/BoyJ (CD45.1^+^) mice were obtained from The Jackson Laboratory. UNC93B1 mutant (3d) mice were generated by Dr. Bruce Beutler at The Scripps Research Institute, La Jolla, California [19]. TLR3, TLR7, TLR9, MyD88 and TRIF deficient mice were provided by Dr. Shizuo Akira (Department of Host Defense, Osaka University, Osaka, Japan). Mice deficient in both MyD88 and TRIF were generated by interbreeding single knockout animals. Except for the TRIF^−/−^ mice, which are F5, all mice used were backcrossed to C57BL/6 background at least for 8 generations. Age (5–8 weeks old) and sex matched groups of wild-type (WT) and knockout mice were used in all experiments. Mice were bred and housed under specific pathogen-free conditions at the University of Massachusetts Medical School animal facilities.

### Parasites

ME-49 tachyzoites were initially obtained by inoculating brain homogenate containing cysts from C57BL/6 mice infected one month earlier onto fibroblast monolayers. After the emergence of tachyzoites, parasites were maintained in human foreskin fibroblast monolayers by weekly passages as described [60]. Luciferase and GFP expressing ME-49 and RH parasites [61] were a kind gift from Dr. Jeroen P. J. Saeij (MIT, Cambridge, MA).

### *In vivo* experimental infections

Animals were inoculated intraperitoneally (i.p.) with 25 cysts obtained from brain homogenates of 6–8 weeks infected mice. Mice were monitored for survival or sacrificed at 0, 5 and 8 days post infection in order to collect peritoneal exudate cells and fluid, blood and spleens. Samples from each mouse were individually processed and analyzed.

### Quantitative real-time PCR

Peritoneal exudate cells, spleen, liver, lung and brain from mice at days 0, 3, 5 or 7 after infection were collected and frozen in liquid nitrogen. Total DNA was extracted using the DNeasy Blood and Tissue kit (Qiagen) according to the manufacturer's instructions, and quantified using a NanoDrop Spectrophotometer (Thermo Scientific). Primers used for amplification of *Toxoplasma gondii* B1 gene (Forward: 5′- CTGGCAAATACAGGTGAAATG-3′; Reverse: 5′- GTGTACTGCGAAAATGAATCC-3′) were designed using the PrimerSelect application (Lasergene software suite, DNASTAR). PCR reactions were setup in a final 20 µl volume using 5 ng of total tissue DNA, 200 ng of each primer and 1x of the iQ SYBR Green Supermix (BioRad). Quantitative RT-PCR analysis was performed on a DNA Engine Opticon 2 Real-Time Cycler (MJ Research). Specificity of amplification was assessed for each sample by melting curve analysis. Non-infected samples gave no signal. Relative quantification was performed using standard curve analysis of purified parasite DNA, and results were expressed as pg of parasite DNA per mg of total tissue DNA.

### Flow cytometry

Cells were stained for 30 min with conjugated antibodies against the surface markers CD3, CD4, CD8, CD11b, CD25, CD40, CD69, CD80, CD86, CD154, MHC I or MHC II (eBioscience). For intracellular measurement of cytokines splenocytes were cultivated for 4 h in presence of GolgiPlug (BD Bioscience), surface stained, permeabilized and incubated with Phycoerythrin-anti- IFNγ or TNFα for 30 min. Subsequently cells were washed and analyzed by flow cytometry in an LSRII cytometer (BD Bioscience). Fluorescent cell lines were analyzed without staining. Data were acquired with DIVA software (BD Bioscience) and analyzed with FlowJo (Tree Star).

### Bone marrow transplantation

Recipient B6.SJL-*Ptprc^a^Pepc^b^*/BoyJ and 3d mice were given lethal total body irradiation (900 rads) and reconstituted intravenously with 5–10 million bone marrow cells within 4 h. Marrow cell suspensions were prepared from donor tibial and femoral bones by flushing with phosphate-buffered saline using a 30-gauge needle syringe. Irradiated and reconstituted mice were given 150 mg/ml Sulfamethoxazole and 30 mg/ml N-Trimethoprim in their drinking water for 6 weeks. Thereafter, they were switched to sterile drinking water, thus ensuring that the antibiotic treatment would not affect the ensuing experimental infection with *T. gondii*. Mice were used for experimental infection or for analysis of chimerism 7–9 weeks after transplant. Animals showed full reconstitution of lymphoid and myeloid cell populations as determined by flow cytometric analysis (not shown).

### Spleen cell culture

Spleens were homogenized, RBC lysed (Red Blood Cell Lysis buffer, Sigma), and splenocytes were re-suspended in complete RPMI medium. Cells were cultured at 5×10^6^/well in 24-well tissue culture plates in absence of exogenous stimuli, and supernatants were collected 24 h later.

### Cytokine and NO measurement

Splenocyte, macrophage culture supernatants or peritoneal exudates were assayed for pro-inflammatory cytokines with DuoSet ELISA kits from R&D Systems according to the manufacturer's instructions. Reactive nitrogen intermediates were measured by the Griess reaction of nitrites accumulated in the supernatants [62] with chemicals from Sigma. Cytokines present in mouse serum were assayed using the BD Cytometric Bead Assay (CBA) Mouse Inflammation kit according to the manufacturer's instructions.

### Immunoblot analysis

Splenocytes collected from either non-infected animals or from mice infected for 5 or 8 days were lysed, the extract re-suspended in Laemmli sample buffer [63] and boiled for 5 min at 95°C. Samples were separated by 10% SDS-PAGE and were transferred onto nitrocellulose membranes [64]. Blots were incubated with mouse monoclonal antibodies against STAT-1 (C-terminus) or STAT-1P (BD Biosciences) and subsequently incubated with HRP-conjugated anti-mouse IgG (Bio-Rad). Membranes were then incubated with HRP substrate (enhanced chemiluminescence substrate; Amersham Biosciences) and developed by exposure to film (Hyperfilm; Amersham Biosciences).

### Cell culture

Bone marrow-derived macrophages (BMDMs) were isolated as described [65], and were cultured in RPMI medium supplemented with 25 mM Hepes, 10 mM L-glutamine, 100 U/ml Penicillin-Streptomycin, 50 µM 2-mercaptoethanol (Sigma) and 10% FCS (Hyclone). Immortalized macrophage cell lines were generated as described [66], [67]. Briefly, primary bone marrow cells were incubated in L929 mouse fibroblast-conditioned medium for 3–4 days for the induction of macrophage differentiation. Subsequently, cells were infected with J2 recombinant retrovirus carrying *v-myc* and *v-raf(mil)* oncogenes [68]. Growth factors were removed from the culture medium and cells were maintained until they were growing in the absence of conditioning medium. Macrophages phenotype was verified by surface expression of the markers CD11b (M1/70, BD Pharmigen) and F4/80 (BM8, eBioscience) as well as a range of functional parameters, including responsiveness to Toll-like ligands. Macrophage cell lines were generated from wild-type (C57BL/6), 3d and TLR9-deficent mice. Cells were treated with the indicated stimuli or infected in a multiplicity of infection (MOI) of 5, and supernatants were collected 24 h later.

### Fluorescent DNA constructs

Murine UNC93B1 (BC018388) was C-terminally fused with YFP and cloned into pcDNA3 (Invitrogen). The point mutant UNC93B1 (H412R) was generated by sequential PCR with primers carrying the point mutation CAC (His) to CGC (Arg). YFP-tagged UNC93B1 wild type and H412R were then cloned into a retroviral vector that was modified from the original pCLXSN backbone from Imgenex (Fig. S6). All of the constructs were verified by sequencing.

### Viral transduction of immortalized macrophages

Recombinant retroviruses were produced as described [69]. Briefly, human embryonic kidney (HEK293T) cells were co-transfected with the vectors encoding YFP-tagged UNC93B1 and plasmids carrying the retroviral *gag-pol* genes and the envelope protein VSV-G using the GeneJuice transfection reagent (Novagen) according to the manufacturer's instructions. The virus containing supernatants were filtered and used to infect immortalized 3d or TLR9-deficient cells.

### *In vitro* infection

ME-49 tachyzoites were stained with the Cell Tracker Red CMTPX (Invitrogen) according to the manufacturer's instruction. Immortalized macrophages were infected in a multiplicity of infection (MOI) of 3. After 2 h, unbound parasites were washed off and live cells were imaged by confocal microscopy at 37°C. Acidic intracellular compartments were stained with the acidophilic lysomotropic dye LysoTracker Blue (Invitrogen). To study the kinetics of parasite growth, BMDMs were infected with GFP-expressing ME-49 tachyzoites in a MOI of 1. After 32 hours cells were fixed and processed for immunofluorescent staining as described below. Number of GRA7-positive vacuoles per cell, and of parasites per vacuole, were evaluated in 10 randomly selected microscopic fields, and at least 100 vacuoles were counted per sample. In order to quantify number of vacuoles positive for interferon-induced GTPases (IRGs), immortalized macrophages were induced overnight with 200 U/ml IFNγ before infection. Cells were fixed and processed for immunofluorescence. Intracellular parasites were identified by GRA7 staining, and the percentage of IRG-positive vacuoles was determined after analysis of 200 vacuoles.

### Cytotoxicity assays

Murine immortalized macrophages were stimulated with IFNγ (BD Pharmigen) at 10–100 U/ml as indicated for 24 h prior to infection while control cultures were left untreated. Cells were then infected with ME-49 tachyzoites at a multiplicity of infection of 1. After 24 h of incubation, cultures were labeled with 1 µCi/well [^3^H]-uracil for additional 24 h. The amount of incorporated uracil was determined by liquid scintillation counting [70]. Alternatively, immortalized macrophages were infected with luciferase-expressing parasites. After 48 h samples were lysed, cell lysates were mixed with a Luciferase reporter assay system substrate (Promega) and luciferase units calculated by normalizing the raw luminescence values to the background from non-infected cells.

### Immunofluorescence

Cells were fixed with paraformaldehyde 4% for 15 min at room temperature, washed with PBS, permeabilized with saponin 0.1% in PBS for 10 min and incubated with primary antibodies for 1 h. Subsequently cells were washed and incubated with Alexa conjugated secondary antibodies for an additional 1 h. Host and parasite DNA was stained with DAPI. Slides were mounted with Gel-mount anti-fading reagent (EMS) and analyzed by confocal microscopy. Primary antibodies used: α-GRA7 antiserum (a kind gift from Dr. George Yap, New Jersey Medical School, Newark, NJ), rabbit α-Irga6 antiserum 165°3 [71], rabbit anti-Irgb6 antiserum 141/1 (raised against bacterial purified full length protein, unpublished), α-Irgm1 (Santa Cruz Biotechnology), and α-Irgm3 (BD Pharmigen). Secondary antibodies: Alexa 633 goat α-rabbit, Alexa 488 donkey α-goat and Alexa 488 goat α-mouse conjugated antibodies (Invitrogen).

### Confocal microscopy

We used an inverted Axiovert 100-M microscope equipped with a Zeiss LSM 510 META scanning unit and a 1.4 NA 63x plan apochromat objective (Zeiss), and an inverted Leica LSM TSC SP2 AOBS. Cells were cultured on glass-bottom 35-mm tissue-culture dishes (Matek). Dual or triple color images were acquired by consecutive scanning with only one laser line active per scan to avoid cross-excitation.

### Statistics

The statistical significance of the differences in the means of experimental groups was determined by one-way or two-way ANOVA analysis and Bonferroni post-test using GraphPad Prism 5.0a Software.

## Supporting Information

Figure S1 Unimpaired cytokine production in nucleotide-sensing TLR deficient mice infected with *T. gondii*. Levels of IL-6, MCP-1, IFNγ, and TNFα were measured in sera of mice at 8 days after infection employing the BD Cytometric Bead Assay (CBA) Mouse Inflammation Kit. IL-12p40 levels in sera of mice at 0 and 8 days post-infection were measured by ELISA.
(0.60 MB TIF)

Figure S2 Cellular analyses of UNC93B1 chimeric mice. (A) Flow cytometry analysis of peripheral blood cells isolated from chimeric mice. Cells were stained with fluorescent antibodies anti-CD45.1 and anti-CD45.2. (B) TNFα and RANTES production by splenocytes collected from non-transplanted control C57BL/6 and 3d mice or chimeric animals. Splenocytes were stimulated overnight with the indicated stimuli, and levels of cytokines measured in culture supernatants by ELISA.
(1.06 MB TIF)

Figure S3 IFNγ responsiveness is not altered in cells from 3d mice. (A) Levels of IL-6, IL-12p40 and TNFα in supernatants of bone marrow-derived macrophages isolated from C57BL/6 or 3d mice cultured for 24 h in medium alone, or in the presence of IFNγ (100 U/ml) and/or LPS (10 ng/ml). (B) Flow cytometry analysis of bone marrow-derived macrophages isolated from C57BL/6 or 3d mice and cultured as in A. Data are representative of two experiments yielding similar results (error bars, s.e.m.).
(0.59 MB TIF)

Figure S4 UNC93B1 do not co-localize with the endolysosomal compartment in resting cells. Confocal microscopy of 3d immortalized macrophages stably expressing wild-type or mutant UNC93B1-YFP. Live cells were stained with LysoTracker Blue and imaged afterwards. Data are representative of four independent experiments.
(5.19 MB PSD)

Figure S5 IFNγ-induced effector mechanisms are functional in 3d cells. (A) Levels of nitrite in supernatants of BMDMs isolated from C57BL/6 or 3d mice cultured for 24 h in medium alone, or in the presence of IFNγ (100 U/ml) and/or LPS (10 ng/ml). (B) BMDMs isolated from C57BL/6 or 3d mice were treated overnight with 100 U/ml IFNγ or left untreated, and the induction of Interferon-related GTPases (IRGs) was analyzed by immunofluorescence. Similarly to 3d cells, untreated wild-type cells were negative for expression of the IRGs tested (not shown). (C) IFNγ induced immortalized macrophages were infected with ME-49 tachyzoites at a MOI of 2.5. After 2 h cells were fixed and stained with the indicated antibodies. Intracellular parasites were identified by GRA7-staining and the percentage of IRG-positive Toxoplasma vacuoles was determined after analysis of 200 vacuoles.
(1.40 MB TIF)

Figure S6 Map of the retroviral vector used to clone wild-type and mutant YFP-tagged UNC93B1.
(0.91 MB TIF)

## References

[1] Hill D, Dubey JP (2002). Toxoplasma gondii: transmission, diagnosis and prevention.. Clin Microbiol Infect.

[2] Denkers EY, Gazzinelli RT (1998). Regulation and function of T-cell-mediated immunity during Toxoplasma gondii infection.. Clin Microbiol Rev.

[3] Akira S, Uematsu S, Takeuchi O (2006). Pathogen recognition and innate immunity.. Cell.

[4] Gazzinelli RT, Denkers EY (2006). Protozoan encounters with Toll-like receptor signalling pathways: implications for host parasitism.. Nat Rev Immunol.

[5] Campos MA, Closel M, Valente EP, Cardoso JE, Akira S (2004). Impaired production of proinflammatory cytokines and host resistance to acute infection with Trypanosoma cruzi in mice lacking functional myeloid differentiation factor 88.. J Immunol.

[6] Scanga CA, Aliberti J, Jankovic D, Tilloy F, Bennouna S (2002). Cutting edge: MyD88 is required for resistance to Toxoplasma gondii infection and regulates parasite-induced IL-12 production by dendritic cells.. J Immunol.

[7] Muraille E, De Trez C, Brait M, De Baetselier P, Leo O (2003). Genetically resistant mice lacking MyD88-adapter protein display a high susceptibility to Leishmania major infection associated with a polarized Th2 response.. J Immunol.

[8] Debierre-Grockiego F, Campos MA, Azzouz N, Schmidt J, Bieker U (2007). Activation of TLR2 and TLR4 by glycosylphosphatidylinositols derived from Toxoplasma gondii.. J Immunol.

[9] Minns LA, Menard LC, Foureau DM, Darche S, Ronet C (2006). TLR9 is required for the gut-associated lymphoid tissue response following oral infection of Toxoplasma gondii.. J Immunol.

[10] Plattner F, Yarovinsky F, Romero S, Didry D, Carlier M (2008). Toxoplasma profilin is essential for host cell invasion and TLR11-dependent induction of an interleukin-12 response.. Cell Host Microbe.

[11] Yarovinsky F, Zhang D, Andersen JF, Bannenberg GL, Serhan CN (2005). TLR11 activation of dendritic cells by a protozoan profilin-like protein.. Science.

[12] Alexopoulou L, Holt A, Medzhitov R, Flavell R (2001). Recognition of double-stranded RNA and activation of NF-kappaB by Toll-like receptor 3.. Nature.

[13] Diebold S, Kaisho T, Hemmi H, Akira S, Reis e Sousa C (2004). Innate antiviral responses by means of TLR7-mediated recognition of single-stranded RNA.. Science.

[14] Heil F, Hemmi H, Hochrein H, Ampenberger F, Kirschning C (2004). Species-specific recognition of single-stranded RNA via toll-like receptor 7 and 8.. Science.

[15] Hemmi H, Takeuchi O, Kawai T, Kaisho T, Sato S (2000). A Toll-like receptor recognizes bacterial DNA.. Nature.

[16] Drennan M, Stijlemans B, Van den Abbeele J, Quesniaux V, Barkhuizen M (2005). The induction of a type 1 immune response following a Trypanosoma brucei infection is MyD88 dependent.. J Immunol.

[17] Bartholomeu D, Ropert C, Melo M, Parroche P, Junqueira C (2008). Recruitment and endo-lysosomal activation of TLR9 in dendritic cells infected with Trypanosoma cruzi.. J Immunol.

[18] Parroche P, Lauw F, Goutagny N, Latz E, Monks B (2007). Malaria hemozoin is immunologically inert but radically enhances innate responses by presenting malaria DNA to Toll-like receptor 9.. Proc Natl Acad Sci U S A.

[19] Tabeta K, Hoebe K, Janssen EM, Du X, Georgel P (2006). The Unc93b1 mutation 3d disrupts exogenous antigen presentation and signaling via Toll-like receptors 3, 7 and 9.. Nat Immunol.

[20] Casrouge A, Zhang S-Y, Eidenschenk C, Jouanguy E, Puel A (2006). Herpes simplex virus encephalitis in human UNC-93B deficiency.. Science.

[21] Brinkmann MM, Spooner E, Hoebe K, Beutler B, Ploegh HL (2007). The interaction between the ER membrane protein UNC93B and TLR3, 7, and 9 is crucial for TLR signaling.. The Journal of Cell Biology.

[22] Kim YM, Brinkmann MM, Paquet ME, Ploegh HL (2008). UNC93B1 delivers nucleotide-sensing toll-like receptors to endolysosomes.. Nature.

[23] Sibley L, Weidner E, Krahenbuhl J (1985). Phagosome acidification blocked by intracellular Toxoplasma gondii.. Nature.

[24] Mordue D, Sibley L (1997). Intracellular fate of vacuoles containing Toxoplasma gondii is determined at the time of formation and depends on the mechanism of entry.. J Immunol.

[25] Joiner K, Fuhrman S, Miettinen H, Kasper L, Mellman I (1990). Toxoplasma gondii: fusion competence of parasitophorous vacuoles in Fc receptor-transfected fibroblasts.. Science.

[26] Gazzinelli RT, Hieny S, Wynn TA, Wolf S, Sher A (1993). Interleukin 12 is required for the T-lymphocyte-independent induction of interferon gamma by an intracellular parasite and induces resistance in T-cell-deficient hosts.. Proc Natl Acad Sci USA.

[27] Gazzinelli RT, Wysocka M, Hayashi S, Denkers EY, Hieny S (1994). Parasite-induced IL-12 stimulates early IFN-gamma synthesis and resistance during acute infection with Toxoplasma gondii.. J Immunol.

[28] Morisaki JH, Heuser JE, Sibley LD (1995). Invasion of Toxoplasma gondii occurs by active penetration of the host cell.. Journal of Cell Science.

[29] Jacobs D, Dubremetz JF, Loyens A, Bosman F, Saman E (1998). Identification and heterologous expression of a new dense granule protein (GRA7) from Toxoplasma gondii.. Mol Biochem Parasitol.

[30] Scharton-Kersten TM, Wynn TA, Denkers EY, Bala S, Grunvald E (1996). In the absence of endogenous IFN-gamma, mice develop unimpaired IL-12 responses to Toxoplasma gondii while failing to control acute infection.. J Immunol.

[31] Brown CR, McLeod R (1990). Class I MHC genes and CD8+ T cells determine cyst number in Toxoplasma gondii infection.. J Immunol.

[32] Goldszmid RS, Bafica A, Jankovic D, Feng CG, Caspar P (2007). TAP-1 indirectly regulates CD4+ T cell priming in Toxoplasma gondii infection by controlling NK cell IFN-gamma production.. J Exp Med.

[33] Reis e Sousa C, Hieny S, Scharton-Kersten T, Jankovic D, Charest H (1997). In vivo microbial stimulation induces rapid CD40 ligand-independent production of interleukin 12 by dendritic cells and their redistribution to T cell areas.. J Exp Med.

[34] Mordue DG, Sibley LD (2003). A novel population of Gr-1+-activated macrophages induced during acute toxoplasmosis.. Journal of Leukocyte Biology.

[35] Sukhumavasi W, Egan CE, Warren AL, Taylor GA, Fox BA (2008). TLR adaptor MyD88 is essential for pathogen control during oral toxoplasma gondii infection but not adaptive immunity induced by a vaccine strain of the parasite.. J Immunol.

[36] Mordue DG, Monroy F, La Regina M, Dinarello CA, Sibley LD (2001). Acute toxoplasmosis leads to lethal overproduction of Th1 cytokines.. J Immunol.

[37] Gavrilescu LC, Denkers EY (2001). IFN-gamma overproduction and high level apoptosis are associated with high but not low virulence Toxoplasma gondii infection.. J Immunol.

[38] Jones TC, Yeh S, Hirsch JG (1972). The interaction between Toxoplasma gondii and mammalian cells. I. Mechanism of entry and intracellular fate of the parasite.. J Exp Med.

[39] Jones TC, Hirsch JG (1972). The interaction between Toxoplasma gondii and mammalian cells. II. The absence of lysosomal fusion with phagocytic vacuoles containing living parasites.. J Exp Med.

[40] Mordue DG, Håkansson S, Niesman I, Sibley LD (1999). Toxoplasma gondii resides in a vacuole that avoids fusion with host cell endocytic and exocytic vesicular trafficking pathways.. Exp Parasitol.

[41] Charron AJ, Sibley LD (2004). Molecular partitioning during host cell penetration by Toxoplasma gondii.. Traffic.

[42] Martens S, Parvanova I, Zerrahn J, Griffiths G, Schell G (2005). Disruption of Toxoplasma gondii parasitophorous vacuoles by the mouse p47-resistance GTPases.. PLoS Pathog.

[43] Sinai AP, Webster P, Joiner KA (1997). Association of host cell endoplasmic reticulum and mitochondria with the Toxoplasma gondii parasitophorous vacuole membrane: a high affinity interaction.. Journal of Cell Science.

[44] Goldszmid RS, Coppens I, Lev A, Caspar P, Mellman I (2009). Host ER-parasitophorous vacuole interaction provides a route of entry for antigen cross-presentation in Toxoplasma gondii-infected dendritic cells.. J Exp Med.

[45] Ewald SE, Lee BL, Lau L, Wickliffe KE, Shi G-P (2008). The ectodomain of Toll-like receptor 9 is cleaved to generate a functional receptor.. Nature.

[46] Zhao YO, Khaminets A, Hunn JP, Howard JC (2009). Disruption of the Toxoplasma gondii parasitophorous vacuole by IFNgamma-inducible immunity-related GTPases (IRG proteins) triggers necrotic cell death.. PLoS Pathog.

[47] Zhao Y, Ferguson DJP, Wilson DC, Howard JC, Sibley LD (2009). Virulent Toxoplasma gondii evade immunity-related GTPase-mediated parasite vacuole disruption within primed macrophages.. J Immunol.

[48] Scharton-Kersten TM, Yap G, Magram J, Sher A (1997). Inducible nitric oxide is essential for host control of persistent but not acute infection with the intracellular pathogen Toxoplasma gondii.. J Exp Med.

[49] Pfefferkorn ER (1984). Interferon gamma blocks the growth of Toxoplasma gondii in human fibroblasts by inducing the host cells to degrade tryptophan.. Proc Natl Acad Sci USA.

[50] Andrade RM, Wessendarp M, Gubbels M-J, Striepen B, Subauste CS (2006). CD40 induces macrophage anti-Toxoplasma gondii activity by triggering autophagy-dependent fusion of pathogen-containing vacuoles and lysosomes.. J Clin Invest.

[51] Ling YM, Shaw MH, Ayala C, Coppens I, Taylor GA (2006). Vacuolar and plasma membrane stripping and autophagic elimination of Toxoplasma gondii in primed effector macrophages.. J Exp Med.

[52] El Hajj H, Lebrun M, Arold ST, Vial H, Labesse G (2007). ROP18 is a rhoptry kinase controlling the intracellular proliferation of Toxoplasma gondii.. PLoS Pathog.

[53] Taylor S, Barragan A, Su C, Fux B, Fentress SJ (2006). A secreted serine-threonine kinase determines virulence in the eukaryotic pathogen Toxoplasma gondii.. Science.

[54] de la Cruz IP, Levin JZ, Cummins C, Anderson P, Horvitz HR (2003). sup-9, sup-10, and unc-93 may encode components of a two-pore K+ channel that coordinates muscle contraction in Caenorhabditis elegans.. J Neurosci.

[55] Greenwald IS, Horvitz HR (1980). unc-93(e1500): A behavioral mutant of Caenorhabditis elegans that defines a gene with a wild-type null phenotype.. Genetics.

[56] Levin JZ, Horvitz HR (1992). The Caenorhabditis elegans unc-93 gene encodes a putative transmembrane protein that regulates muscle contraction.. The Journal of Cell Biology.

[57] Schwab JC, Beckers CJ, Joiner KA (1994). The parasitophorous vacuole membrane surrounding intracellular Toxoplasma gondii functions as a molecular sieve.. Proc Natl Acad Sci USA.

[58] Coppens I, Dunn JD, Romano JD, Pypaert M, Zhang H (2006). Toxoplasma gondii sequesters lysosomes from mammalian hosts in the vacuolar space.. Cell.

[59] Hirschfeld M, Ma Y, Weis J, Vogel S, Weis J (2000). Cutting edge: repurification of lipopolysaccharide eliminates signaling through both human and murine toll-like receptor 2.. J Immunol.

[60] Lock J (1953). Cultivation of Toxoplasma gondii in tissue culture in mammalian cells.. Lancet.

[61] Saeij JPJ, Boyle JP, Grigg ME, Arrizabalaga G, Boothroyd JC (2005). Bioluminescence imaging of Toxoplasma gondii infection in living mice reveals dramatic differences between strains.. Infection and Immunity.

[62] Green L, Wagner D, Glogowski J, Skipper P, Wishnok J (1982). Analysis of nitrate, nitrite, and [15N]nitrate in biological fluids.. Anal Biochem.

[63] Laemmli U (1970). Cleavage of structural proteins during the assembly of the head of bacteriophage T4.. Nature.

[64] Towbin H, Staehelin T, Gordon J (1979). Electrophoretic transfer of proteins from polyacrylamide gels to nitrocellulose sheets: procedure and some applications.. Proc Natl Acad Sci U S A.

[65] Austin P, McCulloch E, Till J (1971). Characterization of the factor in L-cell conditioned medium capable of stimulating colony formation by mouse marrow cells in culture.. J Cell Physiol.

[66] Halle A, Hornung V, Petzold GC, Stewart CR, Monks BG (2008). The NALP3 inflammasome is involved in the innate immune response to amyloid-beta.. Nat Immunol.

[67] Hornung V, Bauernfeind F, Halle A, Samstad EO, Kono H (2008). Silica crystals and aluminum salts activate the NALP3 inflammasome through phagosomal destabilization.. Nat Immunol.

[68] Roberson S, Walker W (1988). Immortalization of cloned mouse splenic macrophages with a retrovirus containing the v-raf/mil and v-myc oncogenes.. Cell Immunol.

[69] Mann R, Mulligan R, Baltimore D (1983). Construction of a retrovirus packaging mutant and its use to produce helper-free defective retrovirus.. Cell.

[70] Pfefferkorn ER, Pfefferkorn LC (1977). Specific labeling of intracellular Toxoplasma gondii with uracil.. J Protozool.

[71] Martens S, Sabel K, Lange R, Uthaiah R, Wolf E (2004). Mechanisms regulating the positioning of mouse p47 resistance GTPases LRG-47 and IIGP1 on cellular membranes: retargeting to plasma membrane induced by phagocytosis.. J Immunol.

