# Supplementary figures and images for: UNC93B1 Mediates Host Resistance to Infection with Toxoplasma gondii
Source: PLoS Pathog. 2010 Aug 26;6(8):e1001071. doi: 10.1371/journal.ppat.1001071 (PMC2928809; doi:10.1371/journal.ppat.1001071)

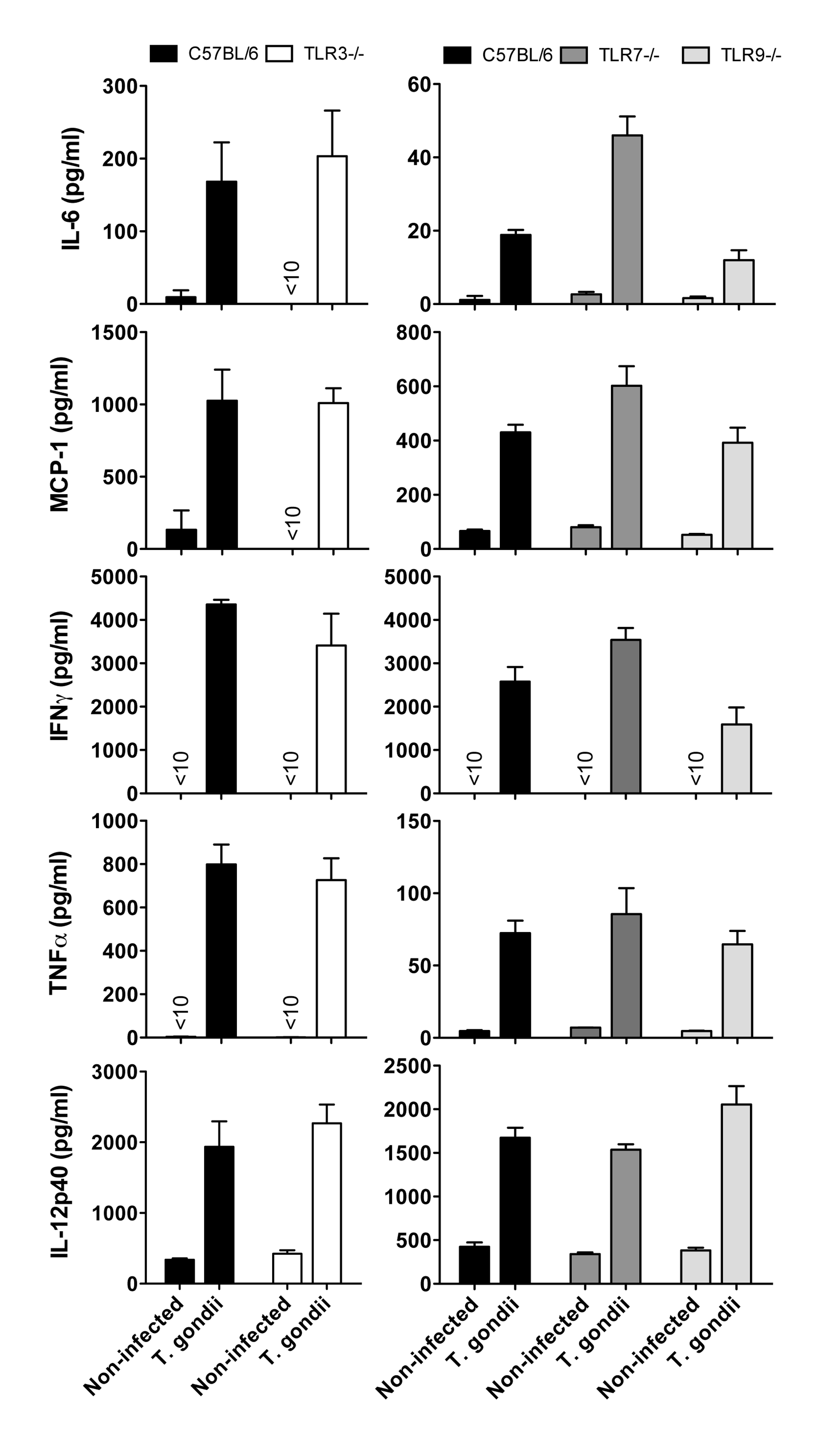

Supplement: Figure S1 — Unimpaired cytokine production in nucleotide-sensing TLR deficient mice infected with T. gondii. Levels of IL-6, MCP-1, IFNγ, and TNFα were measured in sera of mice at 8 days after infection employing the BD Cytometric Bead Assay (CBA) Mouse Inflammation Kit. IL-12p40 levels in sera of mice at 0 and 8 days post-infection were measured by ELISA. (0.60 MB TIF) [file ppat.1001071.s001.tif]

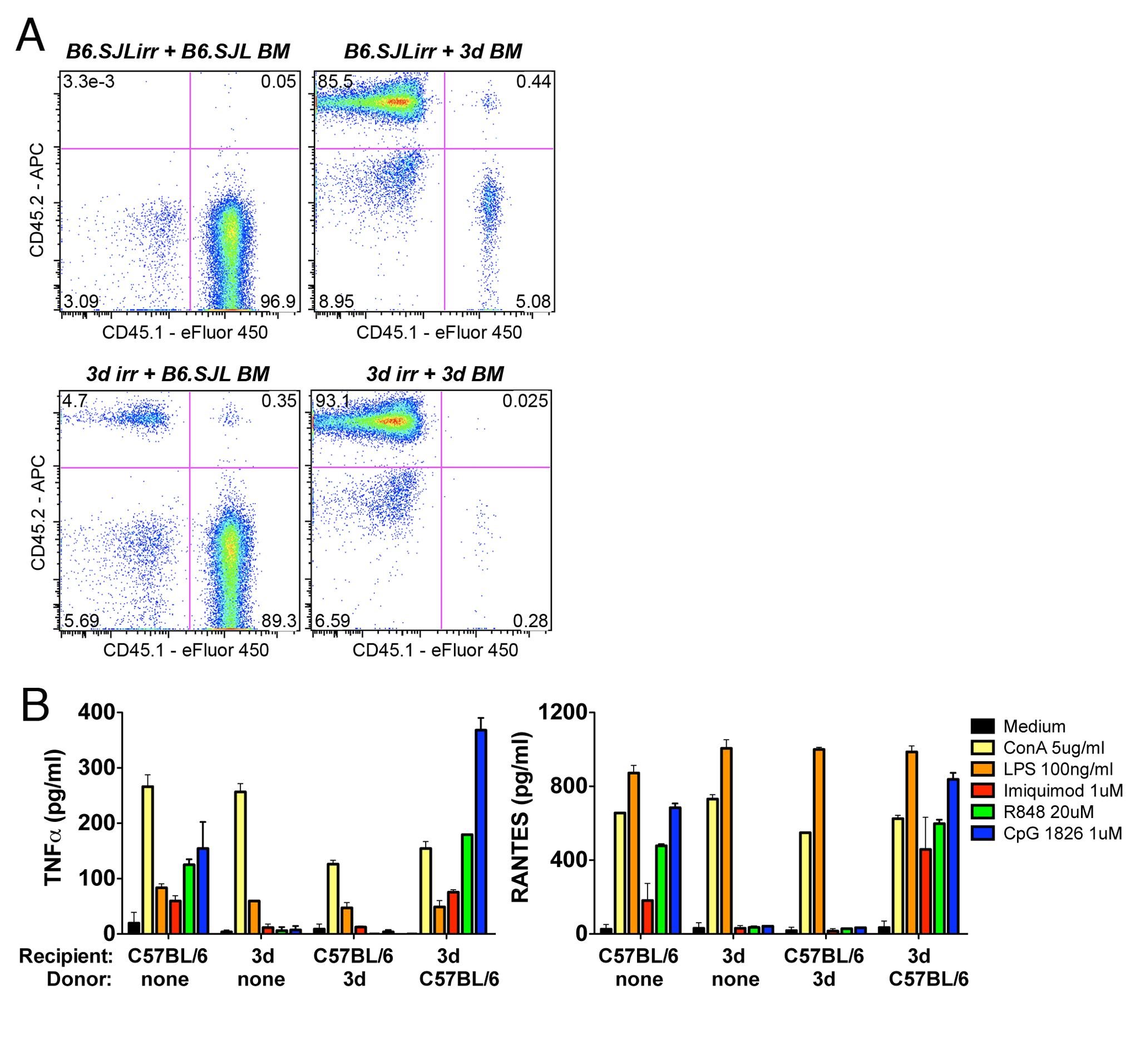

Supplement: Figure S2 — Cellular analyses of UNC93B1 chimeric mice. (A) Flow cytometry analysis of peripheral blood cells isolated from chimeric mice. Cells were stained with fluorescent antibodies anti-CD45.1 and anti-CD45.2. (B) TNFα and RANTES production by splenocytes collected from non-transplanted control C57BL/6 and 3d mice or chimeric animals. Splenocytes were stimulated overnight with the indicated stimuli, and levels of cytokines measured in culture supernatants by ELISA. (1.06 MB TIF) [file ppat.1001071.s002.tif]

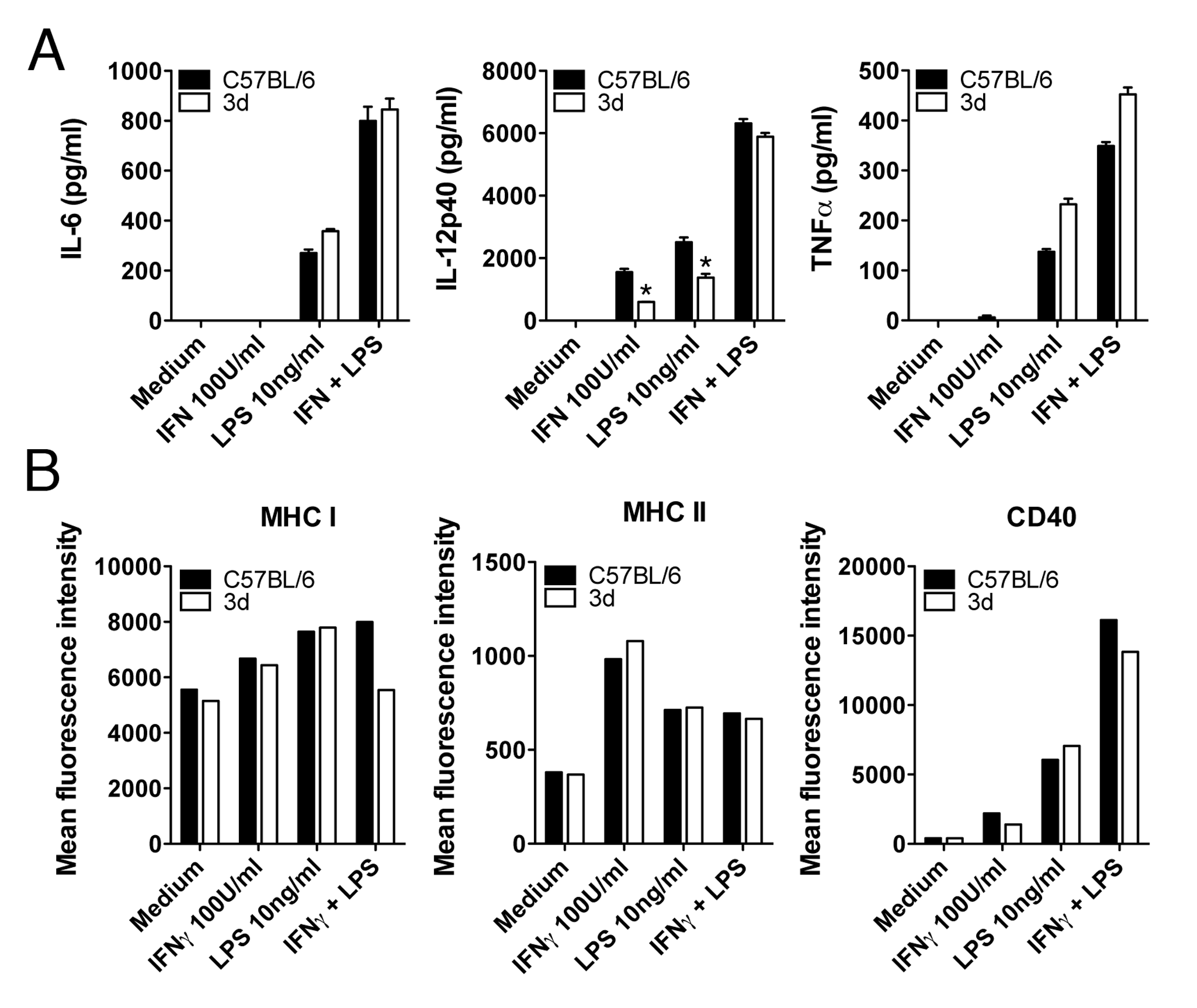

Supplement: Figure S3 — IFNγ responsiveness is not altered in cells from 3d mice. (A) Levels of IL-6, IL-12p40 and TNFα in supernatants of bone marrow-derived macrophages isolated from C57BL/6 or 3d mice cultured for 24 h in medium alone, or in the presence of IFNγ (100 U/ml) and/or LPS (10 ng/ml). (B) Flow cytometry analysis of bone marrow-derived macrophages isolated from C57BL/6 or 3d mice and cultured as in A. Data are representative of two experiments yielding similar results (error bars, s.e.m.). (0.59 MB TIF) [file ppat.1001071.s003.tif]

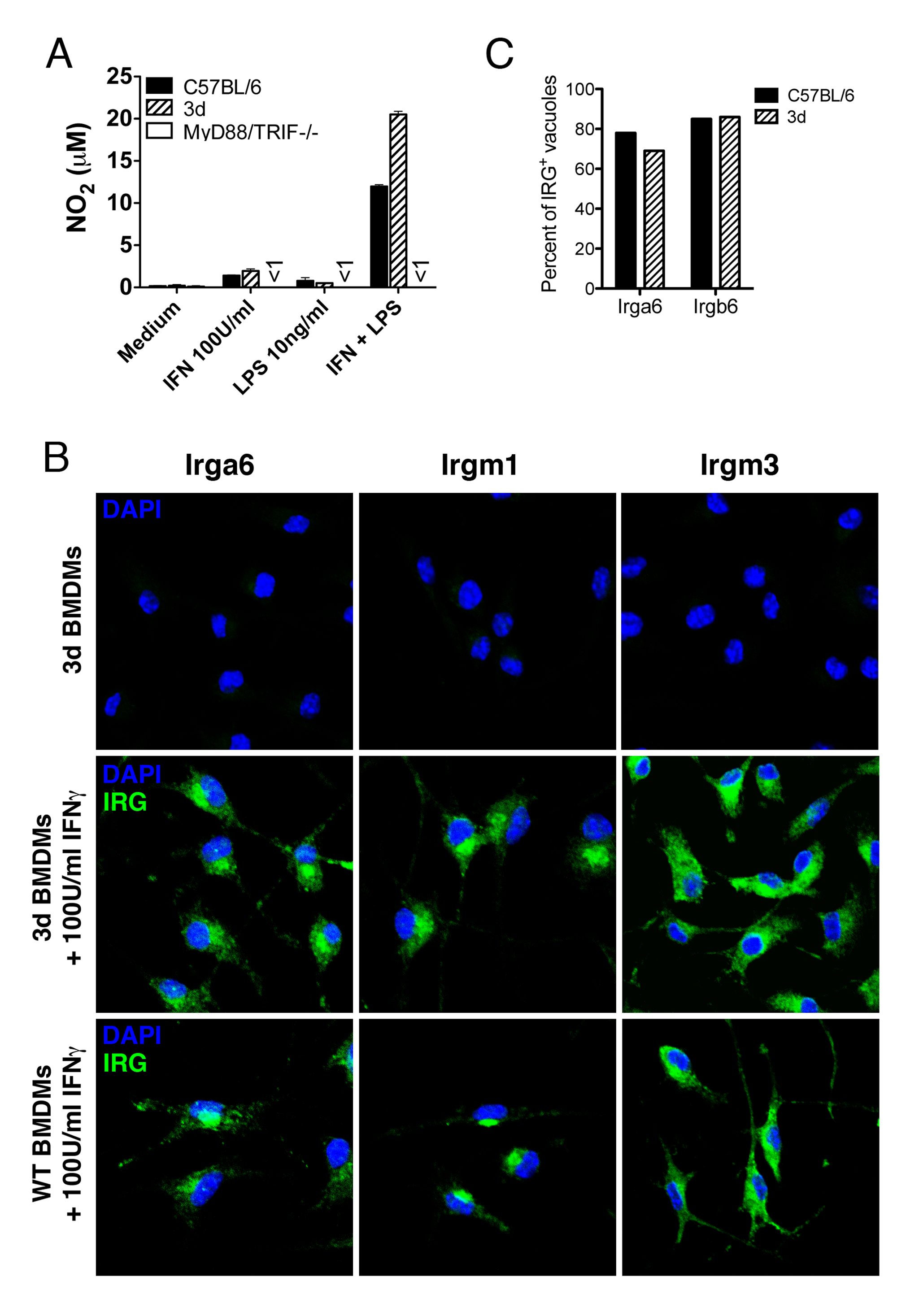

Supplement: Figure S5 — IFNγ-induced effector mechanisms are functional in 3d cells. (A) Levels of nitrite in supernatants of BMDMs isolated from C57BL/6 or 3d mice cultured for 24 h in medium alone, or in the presence of IFNγ (100 U/ml) and/or LPS (10 ng/ml). (B) BMDMs isolated from C57BL/6 or 3d mice were treated overnight with 100 U/ml IFNγ or left untreated, and the induction of Interferon-related GTPases (IRGs) was analyzed by immunofluorescence. Similarly to 3d cells, untreated wild-type cells were negative for expression of the IRGs tested (not shown). (C) IFNγ induced immortalized macrophages were infected with ME-49 tachyzoites at a MOI of 2.5. After 2 h cells were fixed and stained with the indicated antibodies. Intracellular parasites were identified by GRA7-staining and the percentage of IRG-positive Toxoplasma vacuoles was determined after analysis of 200 vacuoles. (1.40 MB TIF) [file ppat.1001071.s005.tif]

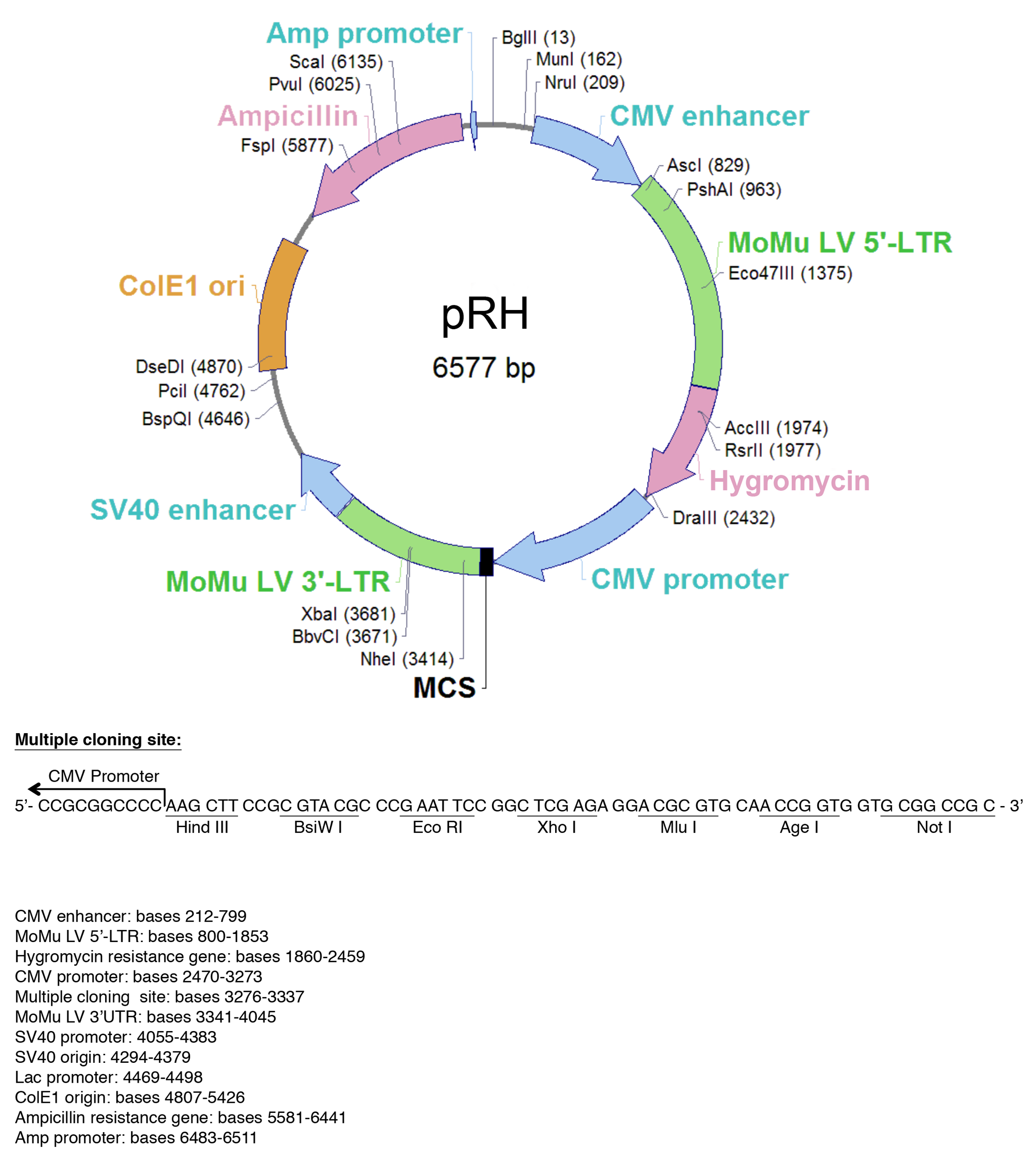

Supplement: Figure S6 — Map of the retroviral vector used to clone wild-type and mutant YFP-tagged UNC93B1. (0.91 MB TIF) [file ppat.1001071.s006.tif]
